# HIV reservoir and premature aging: risk factors for aging-associated illnesses in adolescents and young adults with perinatally acquired HIV

**DOI:** 10.1371/journal.ppat.1012547

**Published:** 2024-09-23

**Authors:** Maria Raffaella Petrara, Elena Ruffoni, Francesco Carmona, Ilaria Cavallari, Sandra Zampieri, Marzia Morello, Paola Del Bianco, Osvalda Rampon, Nicola Cotugno, Paolo Palma, Paolo Rossi, Carlo Giaquinto, Silvia Giunco, Anita De Rossi

**Affiliations:** 1 Immunology and Molecular Oncology Diagnostics, Veneto Institute of Oncology IOV–IRCCS, Padova, Italy; 2 Oncology and Immunology Section, Department of Surgery, Oncology and Gastroenterology, University of Padova, Padova, Italy; 3 Department of Surgery, Oncology and Gastroenterology, University of Padova, Padova, Italy; 4 Department of Biomedical Sciences, University of Padova, Padova, Italy; 5 Clinical Research Unit, Veneto Institute of Oncology IOV-IRCCS, Padova, Italy; 6 Department of Women’s and Children’s Health, Division of Pediatric Infectious Diseases, University of Padova, Padova, Italy; 7 Clinical and Research Unit of Clinical Immunology and Vaccinology, Bambino Gesù Children’s Hospital, IRCCS, Roma, Italy; Vaccine Research Center, UNITED STATES OF AMERICA

## Abstract

Despite receiving antiretroviral therapy (ART), an increasing number of adolescents and young adults with perinatally acquired HIV (PHIVAYA) are at risk of developing premature senescence and aging-associated illnesses, including cancer. Given this concern, it is crucial to assess aging biomarkers and their correlation with the HIV reservoir in order to comprehensively characterize and monitor these individuals. Fifty-five PHIVAYA (median age: 23, interquartile range [IQR]: 20–27 years, and 21 [18–23] years on ART at the time of study sampling) were studied along with 23 age-matched healthy controls. The PHIVAYA exhibited significantly higher percentages of activated, senescent, exhausted CD4 and CD8 T cells, shorter telomeres, reduced thymic output, and higher levels of circulating inflammatory markers (PAMPs, DAMPs, and pro-inflammatory cytokines IL-6, IL-8, and TNFα) as well as denervation biomarkers (neural cell adhesion molecule 1 [NCAM1] and C-terminal Agrin fragment [CAF]), compared to controls. HIV-DNA levels positively correlated with activated, senescent, exhausted CD4 and CD8 T cells, circulating biomarkers levels, and inversely with regulatory T and B cells and telomere length. According to their viremia over time, PHIVAYA were subgrouped into 14 Not Suppressed (NS)-PHIVAYA and 41 Suppressed (S)-PHIVAYA, of whom 6 who initiated ART within one year of age and maintained sustained viral suppression overtime were defined as Early Suppressed (ES)-PHIVAYA and the other 35 as Late Suppressed (LS)-PHIVAYA. ES-PHIVAYA exhibited significantly lower HIV-DNA reservoir, decreased percentages of senescent and exhausted CD4 and CD8 T cells, reduced levels of circulating inflammatory and denervation biomarkers, but longer telomere compared to LS- and NS-PHIVAYA. They differed significantly from healthy controls only in a few markers, including higher percentages of regulatory T and B cells, and higher levels of DAMPs. Overall, these results underscore the importance of initiating ART early and maintaining viral suppression to limit the establishment of the viral reservoir and to counteract immune and cellular premature aging. These findings also suggest new approaches for minimally invasive monitoring of individuals at high risk of developing premature aging and age-related illnesses.

## Author summary

The introduction of combined antiretroviral therapy has led to the transition of HIV perinatally infected children into adolescence and young adulthood, thereby increasing their susceptibility to aging-related diseases, including cancer. While previous studies have examined aging markers in individuals living with HIV, no study has comprehensively integrated multifaceted features of aging in adolescents/young adults with perinatally acquired HIV (PHIVAYA). In this study, we conducted a comprehensive evaluation of a multiparametric aging profile, including immunophenotypic aspects (activated, senescent and exhausted T and B cells), cellular markers (telomere length and thymic output), circulating inflammatory (PAMPs, DAMPs, cytokines) and denervation (CAF and NCAM1) biomarkers. Our findings demonstrate that aging biomarkers in PHIVAYA were altered compared to age-matched controls and were correlated with HIV-DNA reservoir levels. Importantly, PHIVAYA with early and sustained viral suppression exhibited levels of most aging biomarkers comparable to those of controls. The insights from this study have the potential to significantly impact healthcare strategies and improve the well-being of these individuals.

## Introduction

The introduction of combined antiretroviral therapy (ART) has yielded significant benefits, including suppression of HIV replication, restoration of immune function, reduction of HIV-related morbidity and mortality, but it does not fully restore health [1]. The persistence of latent HIV reservoir necessitates lifelong treatment to maintain virus suppression [2]; indeed, despite ART, viral reservoir persistence, mainly due to damage to the gut mucosa and the release of pathogen-associated molecular patterns (PAMPs) like 16S ribosomal (r)DNA, and damage-associated molecular patterns (DAMPs) such as mitochondrial (mt)DNA, induces a chronic state of inflammation/immune activation, which likely leads to biological and immunological senescence, including shorter telomeres and higher percentages of exhausted and senescent immune cells [3]. This progressive accumulation of perturbations is associated with a progressive decline in health, leading to the premature onset of age-related conditions, such as metabolic, cardiovascular, neurological disorders, and cancers [4–6]. It is worth noting that although ART reduced mortality among individuals living with HIV, cancer remains a significant cause of death. Recent years have seen a shift from AIDS-associated tumors, primarily attributable to immunosuppression and interaction with other oncogenic viruses, to non-AIDS-associated tumors which are on the rise as the HIV population ages [7].

Aging involves a remodelling of the immune system, including reduced thymic output and circulating naive T cells, increased frequency of well-differentiated memory CD28- T cells with limited proliferative potential, elevated levels of several pro-inflammatory cytokines, and a decreased CD4/CD8 lymphocyte ratio [1]. Frailty, which encompasses sarcopenia and muscle weakness, is also a hallmark of aging. The neuromuscular junction (NMJ) undergoes deleterious morphological, functional and molecular changes, and ultimately degenerates, releasing soluble isoforms of neural cell adhesion molecule 1 (NCAM1), identified as a marker of denervation [8, 9]. In addition, circulating elevated levels of C-terminal Agrin fragment (CAF), crucial for the formation and stabilization of NMJ, have been identified as a marker of sarcopenia [10].

Limited data were available in this context [11–13]: functional impairment in elderly living with HIV during successful ART was associated with higher CD8 cell activation and IL-6 levels [11], as well as higher CD8 cell senescence and plasma levels of sCD14 [12]. In a study evaluating circulating markers of inflammation in frail or non-frail individuals living with or without HIV, the highest levels of sCD14 and TNF-α has been found in HIV-positive frail individuals [13].

To date, available data concerning children with perinatally acquired HIV infection have shown that the viral reservoir is associated with increased inflammation and immune activation, likely resulting in early immune exhaustion and senescence [14–16]. However, very little is known about biological and immunosenescence in perinatally acquired HIV adolescents and young adults (PHIVAYA) under continuous ART for a long period of time [17–19]. Identifying a multifaceted aging profile, characterized by biological, immunological and denervation markers and their correlation with the viral reservoir, can be useful in designing new approaches for minimally invasive monitoring of individuals at high risk of developing premature age-related illnesses, including cancers.

## Results

### 1. Characteristics of the studied populations

A total of 78 adolescents and young adults were included in this study: 55 PHIVAYA and 23 healthy controls with a median [interquartile range-IQR] age of 23 [20–27] and 19 [18–27] years, p = 0.107, respectively. PHIVAYA initiated ART at a median age of 3.5 [0.9–5.9] years and had been on ART for a median duration of 21 [18–23] years at the time of study sampling.

**Fig 1:**
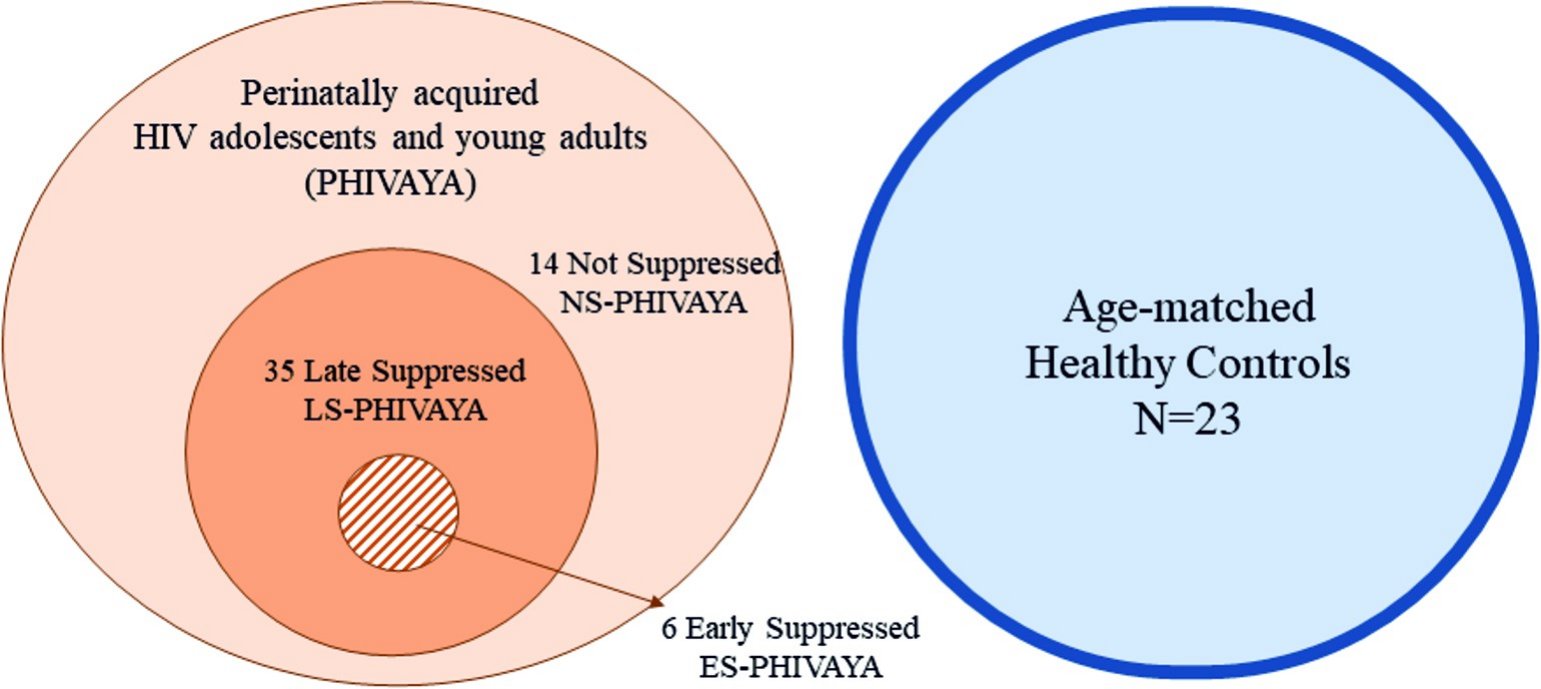
Schematic representation of the studied populations at the time of study sampling. Characteristics of PHIVAYA subgroups (NS-PHIVAYA, LS-PHIVAYA and ES-PHIVAYA) and healthy control group.

As detailed in Materials and Methods, according to their plasmaviremia over time, the 55 PHIVAYA were subgrouped into: 14 Not Suppressed (NS)-PHIVAYA, characterized by transient periods of detectable viremia (>1000 copies/ml), and 41 Suppressed (S)-PHIVAYA, with undetectable HIV-RNA plasma levels (<50 copies/ml) and with no more than one annual viral blip of HIV-RNA (from 50 to 400 HIV-RNA copies/ml) for at least the last 10 years of follow-up. Within the S-PHIVAYA group, 6 individuals were classified as Early Suppressed (ES)-PHIVAYA, characterized by undetectable HIV-RNA plasma levels (<50 copies/ml) achieved after viral suppression within 12 months of ART initiation, and the other 35 as Late Suppressed (LS)-PHIVAYA. The characteristics of the studied populations at the time of study sampling are shown in Fig 1 and Table 1.

**Table 1:** Characteristics of the studied populations at the time of study sampling.

Characteristics Median [IQR]	NS-PHIVAYA (N = 14)	LS-PHIVAYA (N = 35)	ES-PHIVAYA (N = 6)	Healthy controls (N = 23)
gender (M/F)	7/7	13/22	3/3	17/6
Age (years)	24 [21–27]	24 [19–27]	21 [19–22]	19 [18–27]
Age at ART initiation (years)	5 [3–8]	4 [1–6]	0.3 [0.1–0.8]	-
Time on ART (years)	21 [17–22]	22 [17–23]	21 [19–21]	-
Combination of ART:				
NRTI/PI	2	21	2	-
NRTI/NNRTI	-	6	4	-
NRTI/NNRTI/PI	1	2	-	-
NRTI/ CCR5 inhibitors	2	2	-	-
NRTI/PI/CCR5 inhibitors	6	2	-	-
NRTI/NNRTI/CCR5 inhibitors	-	1	-	-
NRTI/NNRTI/PI/CCR5 inhibitors	3	-	-	-
Comorbidities*	4/14	17/35	1/6	-

IQR: interquartile range; ART: antiretroviral therapy; NRTI: nucleoside reverse transcriptase inhibitors; NNRTI: non-nucleoside reverse transcriptase inhibitors; PI: protease inhibitors.

* lipodystrophy, dyslipidemia, hypercholesterolemia, osteopenia, HIV encephalopathy, chronic renal failure, arterial hypertension, psoriasis, cone-rod dystrophy, polycystic ovary, non-alcoholic fatty liver disease, diabetes type I, multifocal progressive leukoencephalopathy.

### 2. Immunological and cellular aging biomarkers of the PHIVAYA subgroups

First of all, we compared the immunological and cellular aging biomarkers between S-PHIVAYA and NS-PHIVAYA (Table 2). The two subgroups showed significant differences: the S-PHIVAYA exhibited higher percentage of CD4 cells, lower percentage of CD8 cells, resulting in a higher CD4/CD8 ratio, lower percentages of activated CD4 cells, senescent CD4 and CD8 cells, and exhausted CD8 compared to the NS-PHIVAYA (Table 2). In addition, S-PHIVAYA had significantly longer telomeres (RTL) and higher thymic output (TREC) than NS-PHIVAYA (Table 2).

**Table 2:** Immunological and cellular aging biomarkers in Suppressed (S) and Not Suppressed (NS) PHIVAYA.

Parameters Median [IQR]	PHIVAYA (N = 55)	S-PHIVAYA (N = 41)	NS-PHIVAYA (N = 14)	p-value*
%CD4	35.6 [28.2–38.9]	37.0 [29.8–39.7]	30.9 [24.4–35.5]	**0.003**
%CD8	31.7 [25.3–40.2]	30.3 [24.7–36.8]	36.8 [31.2–42.2]	**0.008**
CD4/CD8	1.0 [0.8–1.4]	1.2 [0.9–1.7]	0.8 [0.6–1.1]	**0.006**
CD4 activation (%CD3+CD4+CD38+HLA-DR+)	1.4 [1.0–2.5]	1.2 [1.0–2.1]	2.3 [1.3–3.1]	**0.028**
CD8 activation (%CD3+CD8+CD38+HLA-DR+)	1.3 [0.7–2.1]	1.2 [0.7–1.8]	1.7 [0.7–2.5]	0.297
B activation (%CD19+CD10-CD21-CD27+)	8.0 [6.0–10.9]	7.6 [6.0–10.4]	9.7 [5.7–13.4]	0.979
CD4 senescence (%CD3+CD4+CD27-CD45RA+CD28-CD57+)	12.5 [5.9–22.2]	8.1 [5.3–18.2]	20.6 [16.9–27.9]	**0.000**
CD8 senescence (%CD3+CD8+CD27-CD45RA+CD28-CD57+)	6.5 [3.4–12.8]	5.3 [3.2–10.8]	13.8 [7.5–17.4]	**0.023**
B senescence (%CD19+CD27-IgD-)	10.4 [7.2–14.6]	10.5 [8.4–14.6]	9.1 [6.3–14.3]	0.212
CD4 exhaustion (%CD3+CD4+ TIGIT+)	3.9 [2.8–7.4]	3.7 [2.0–6.9]	5.3 [3.7–8.0]	0.710
CD8 exhaustion (%CD3+CD8+TIGIT+)	17.8 [13.6–33.6]	15.6 [11.9–20.5]	41.6 [32.5–53.0]	**0.000**
T-regs (%CD4+CD25+CD127-FoxP3+)	11.8 [9.4–19.5]	12.2 [9.5–19.8]	11.6 [9.4–15.4]	0.750
B-regs (%CD19+CD24++CD38++)	3.3 [2.4–4.4]	3.3 [2.5–4.7]	3.5 [2.5–4.1]	0.505
TREC copies/10^6^ PBMC	379 [213–692]	425 [289–809]	91 [38–251]	**0.000**
RTL	1.2 [1.1–1.3]	1.2 [1.1–1.3]	1.1 [1.1–1.2]	**0.012**

* Adjusted by age, time on ART and time of ART initiation.

**Fig 2:**
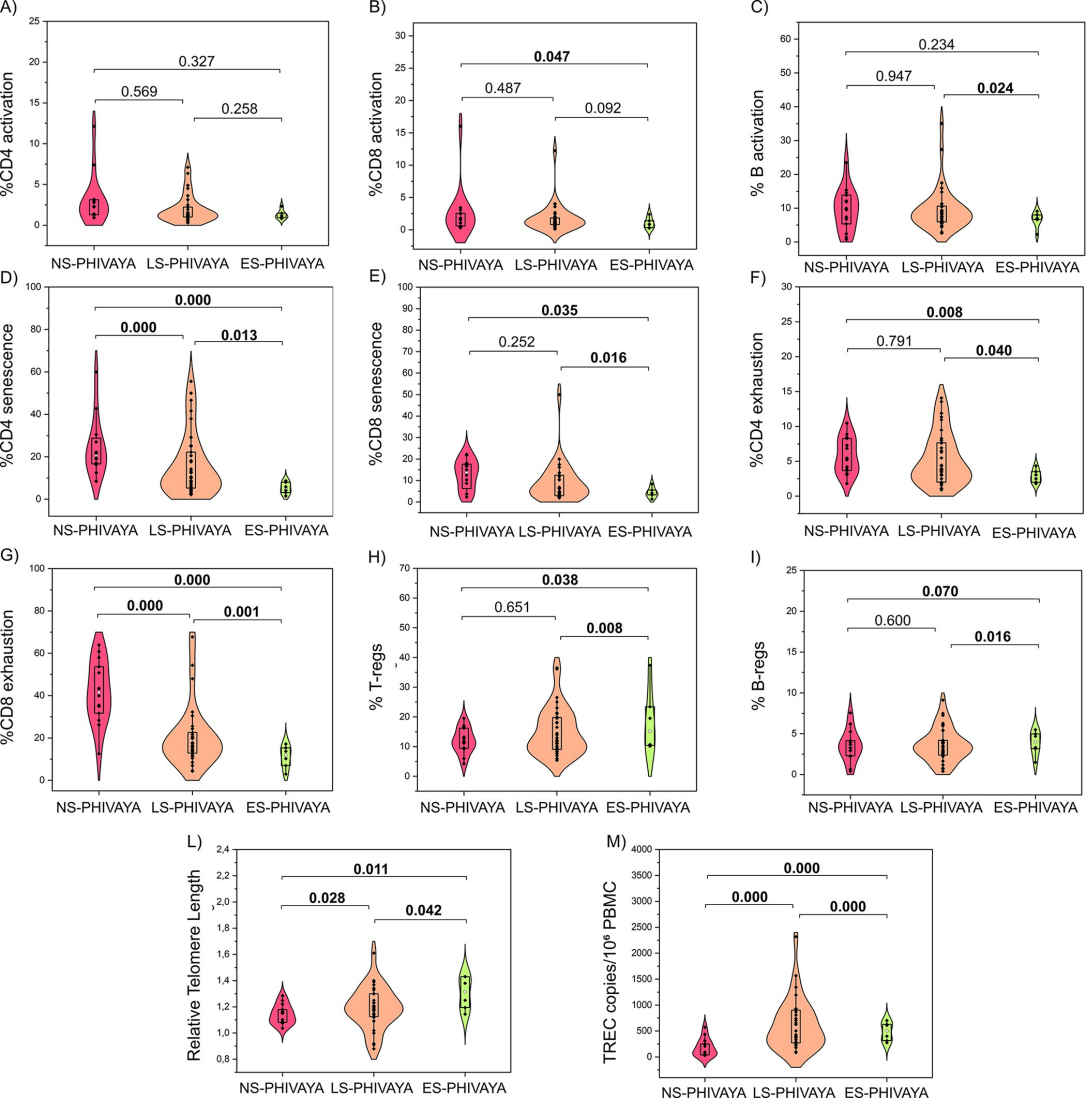
Comparison of immune and cellular markers of aging among PHIVAYA subgroups. Comparison of levels of activated CD4 (A), CD8 (B) and B (C) cells; senescent CD4 (D) and CD8 (E) cells; exhausted CD4 (F) and CD8 (G) cells; regulatory T (T-regs) (H), and B (B-regs) cells (I), relative telomere length (L), and thymic output (TREC) (M) among PHIVAYA subgroups; p-values were adjusted for age, time on ART and time of ART initiation.

Within the S-PHIVAYA subgroup, the 6 ES-PHIVAYA displayed lower percentage of CD8 cells, higher CD4/CD8 ratio, lower percentages of senescent and exhausted CD4 and CD8 cells and higher percentages of T and B regulatory cells compared to LS-PHIVAYA (Table 3). Notably, ES-PHIVAYA also exhibited significantly longer telomeres and higher TREC levels than LS-PHIVAYA (Table 3). Overall, the ES-PHIVAYA had lower levels of immune activated, senescent, and exhausted CD4 and CD8 cells, highest levels of T and B regulatory cells and thymic output, and longer telomeres compared to the other PHIVAYA subgroups (Fig 2).

**Table 3:** Immunological and cellular aging biomarkers of Early Suppressed (ES)- and Late Suppressed (LS)-PHIVAYA.

Parameters Median [IQR]	S-PHIVAYA (N = 41)	ES-PHIVAYA (N = 6)	LS-PHIVAYA (N = 35)	p-value*
%CD4	37.0 [29.8–39.7]	37.9 [36.4–38.7]	36.9 [28.6–39.9]	0.886
%CD8	30.3 [24.7–36.8]	26.9 [20.2–30.0]	33.0 [24.8–40.2]	**0.023**
CD4/CD8	1.2 [0.9–1.7]	1.4 [1.3–2.0]	1.0 [0.8–1.7]	**0.021**
CD4+ activation (%CD3+CD4+CD38+HLA-DR+)	1.2 [1.0–2.1]	1.1 [1.0–1.4]	1.3 [1.0–2.2]	0.258
CD8+ activation (%CD3+CD8+CD38+HLA-DR+)	1.2 [0.7–1.8]	0.8 [0.3–1.4]	1.2 [0.8–1.8]	0.092
B activation (%CD19+CD10-CD21-CD27+)	7.6 [6.0–10.4]	6.9 [6.7–7.8]	8.3 [6.0–10.5]	**0.024**
CD4+ senescence (%CD3+CD4+CD27-CD45RA+CD28-CD57+)	8.1 [5.3–18.2]	5.0 [3.4–7.5]	10.0 [5.3–21.4]	**0.013**
CD8+ senescence (%CD3+CD8+CD27-CD45RA+CD28-CD57+)	5.3 [3.2–10.8]	3.8 [3.3–5.2]	6.4 [3.1–12.3]	**0.016**
B senescence (%CD19+CD27-IgD-)	10.5 [8.4–14.6]	12.0 [9.4–14.0]	10.3 [8.3–14.6]	0.879
CD4+ exhaustion (%CD3+CD4+TIGIT+)	3.7 [2.0–6.9]	2.8 [2.1–3.4]	3.8 [2.3–7.6]	**0.040**
CD8+ exhaustion (%CD3+CD8+TIGIT+)	15.6 [11.9–20.5]	12.0 [7.8–14.9]	16.0 [13.2–22.1]	**0.001**
T-regs (%CD4+CD25+CD127-FoxP3+)	12.2 [9.5–19.8]	15.1 [10.5–22.5]	12.2 [9.1–19.7]	**0.008**
B-regs (%CD19+CD24++CD38++)	3.3 [2.5–4.7]	4.0 [3.2–4.9]	3.3 [2.4–4.2]	**0.016**
TREC copies/10^6^PBMC	425 [289–809]	505 [336–624]	425 [280–884]	**0.000**
RTL	1.2 [1.1–1.3]	1.3 [1.2–1.4]	1.2 [1.1–1.3]	**0.042**

* Adjusted by age, time on ART and time of ART initiation.

### 3. Viral reservoir in the PHIVAYA subgroups

The persistence of the HIV reservoir is contributed by clonal expansion of a few clones containing intact, replication-competent proviruses, but primarily by defective, replication-incompetent proviruses, which may be transcriptionally active [20–22].

**Fig 3:**
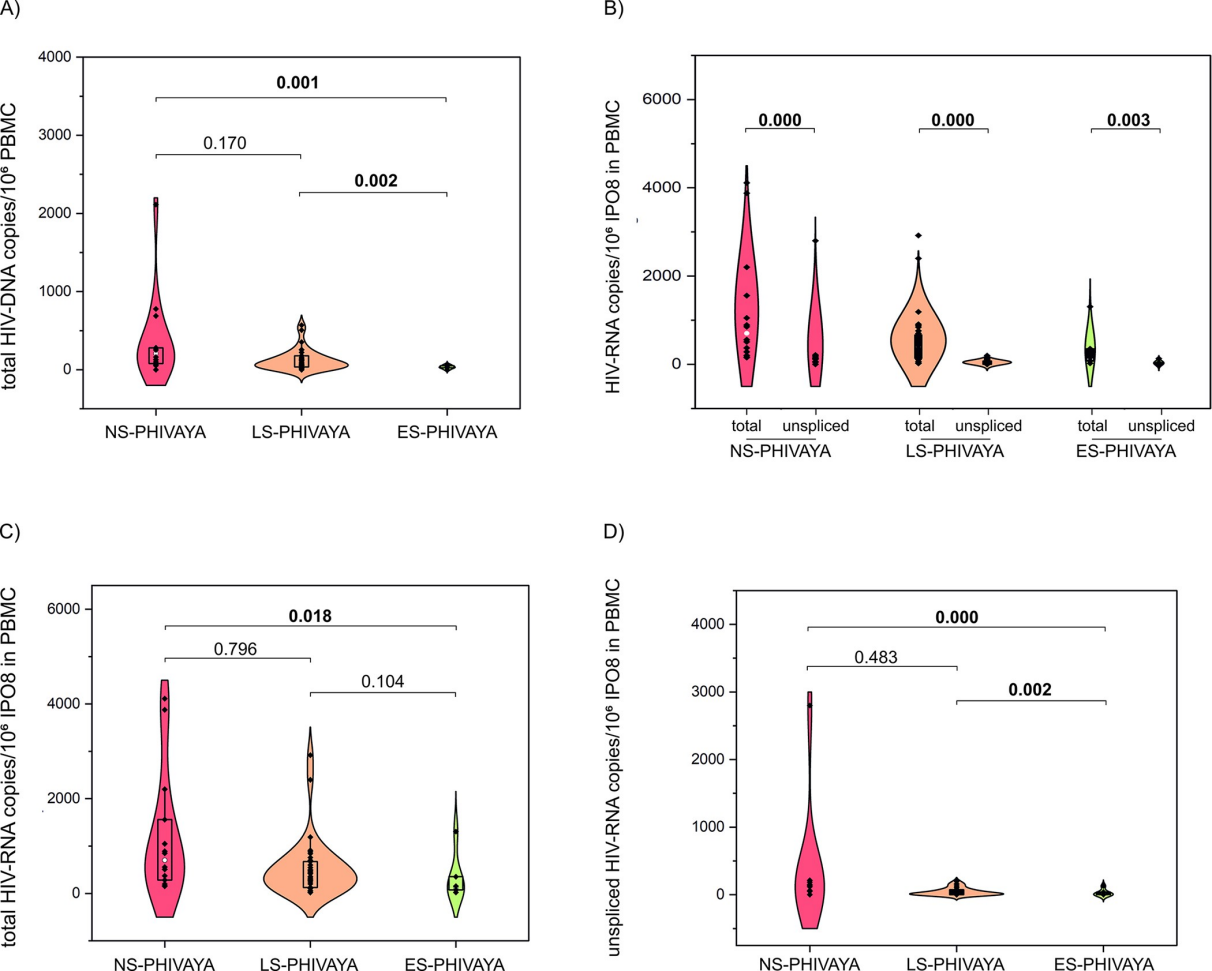
Comparison of HIV-DNA and cell-associated HIV-RNA levels among PHIVAYA subgroups. Comparison of levels of total HIV-DNA (A), total cell-associated HIV-RNA (C), unspliced HIV-RNA (D) and between total and unspliced HIV-RNA (B) among PHIVAYA subgroups; p-values were adjusted for age, time on ART and time of ART initiation.

We measured the total proviral DNA, the total cell-associated HIV-RNA and the unspliced HIV-RNA, which can represent the full-length genomic HIV-RNA. Levels of total HIV-DNA were significantly higher in NS- than in S-PHIVAYA (Table A in S1 Table). Overall, ES-PHIVAYA exhibited lowest levels of total HIV-DNA compared to LS- and NS-PHIVAYA (32 [27–47] *vs* 70 [37–168] *vs* 208 [84–281] copies/10^6^ PBMC) (Fig 3A and Table B in S1 Table).

Levels of unspliced HIV-RNA were significantly lower compared to those of total cell-associated HIV-RNA, not only in ES-PHIVAYA (1 [1–30] *vs* 118 [77–304] copies/10^6^ IPO8 in PBMC; p = 0.003) and LS-PHIVAYA (26 [1–76] *vs* 424 [126–675] copies/10^6^ IPO8 in PBMC; p = 0.000), but also in NS- PHIVAYA (90 [26–158] *vs* 704 [306–1432] copies/10^6^ IPO8 in PBMC; p = 0.000) (Fig 3B).

Within the PHIVAYA subgroups, levels of both total cell-associated and unspliced HIV-RNA were significantly higher in NS- than S-PHIVAYA (Table A in S1 Table). Additionally, levels of both total cell-associated HIV-RNA (Fig 3C and Table B in S1 Table) and unspliced HIV-RNA were significantly lower in ES- than in NS- and LS-PHIVAYA (Fig 3D and Table B in S1 Table).

**Fig 4:**
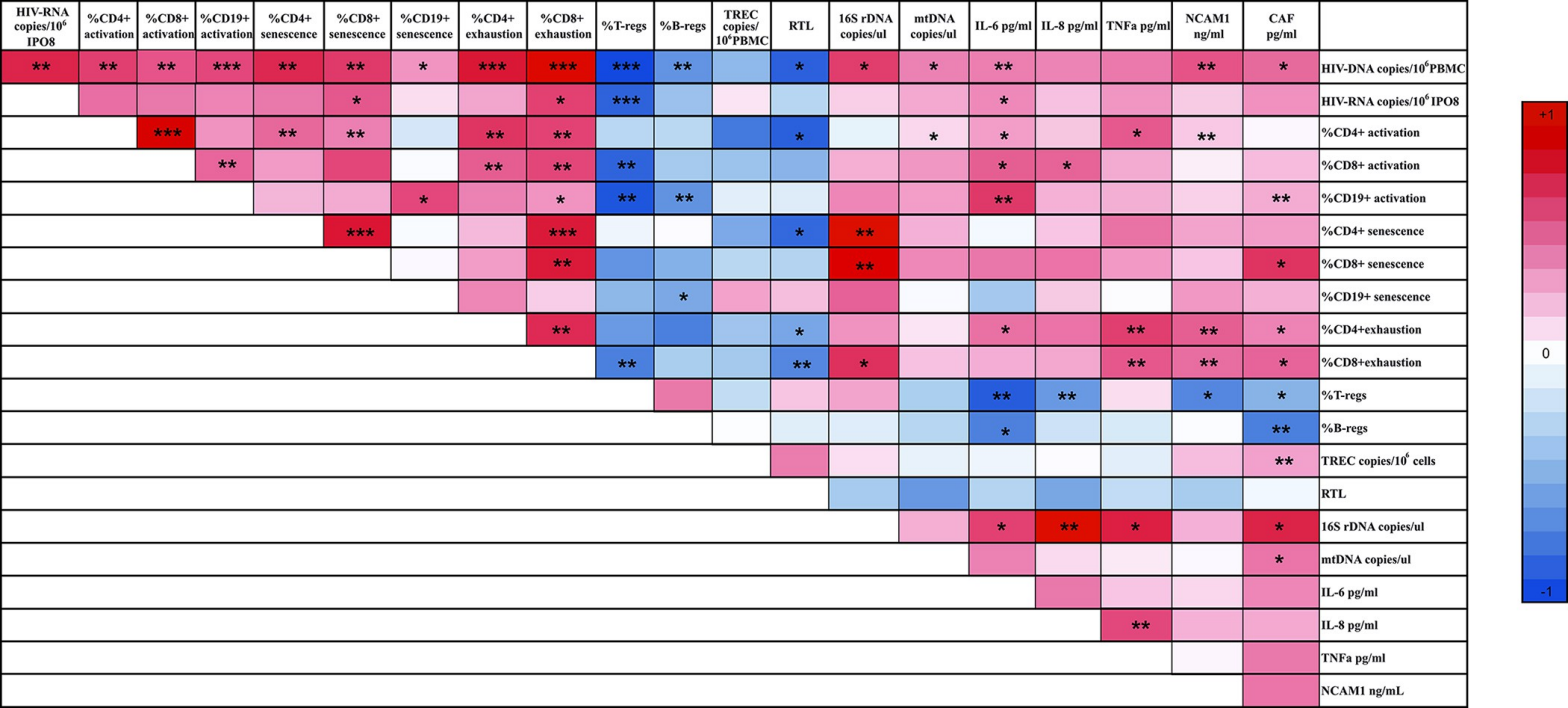
Correlation plot between HIV reservoir and immune and cellular markers in PHIVAYA. Colour scale represents Spearman’s correlation coefficient. Red and blue correspond to positive and negative coefficients, respectively. Asterisks * p< 0.05, ** p<0.01, *** p<0.001.

The entire size of the HIV reservoir was estimated considering levels of the total proviral DNA and levels of total cell-associated HIV-RNA. In the entire cohort, levels of total HIV-DNA and total cell-associated HIV-RNA were positively correlated (r = 0.500, p = 0.001), and their relationships with immunological markers of senescence were detailed in Fig 4 and S2 Table. Specifically, total HIV-DNA levels showed positive correlations with activated CD4, CD8, and B cells, senescent CD4, CD8, and B cells, and exhausted CD4 and CD8 cells. Conversely, HIV-DNA displayed inverse correlations with T and B regulatory cells and telomere length (Fig 4 and S2 Table).

Total cell-associated HIV-RNA tended to be positively correlated with percentages of activated CD4, CD8 and B cells, senescent and exhausted CD8 cells, while inversely correlated with T regulatory cells (Fig 4 and S2 Table).

Remarkably, most of these correlations, in particular for HIV-DNA, remained significant even after adjusting for age, time on ART and time of ART initiation (Fig 4 and S2 Table), suggesting that the HIV *itself* directly impacts immune activation and senescence, rather than therapeutic treatment.

### 4. Viral reservoir and circulating markers

**Fig 5:**
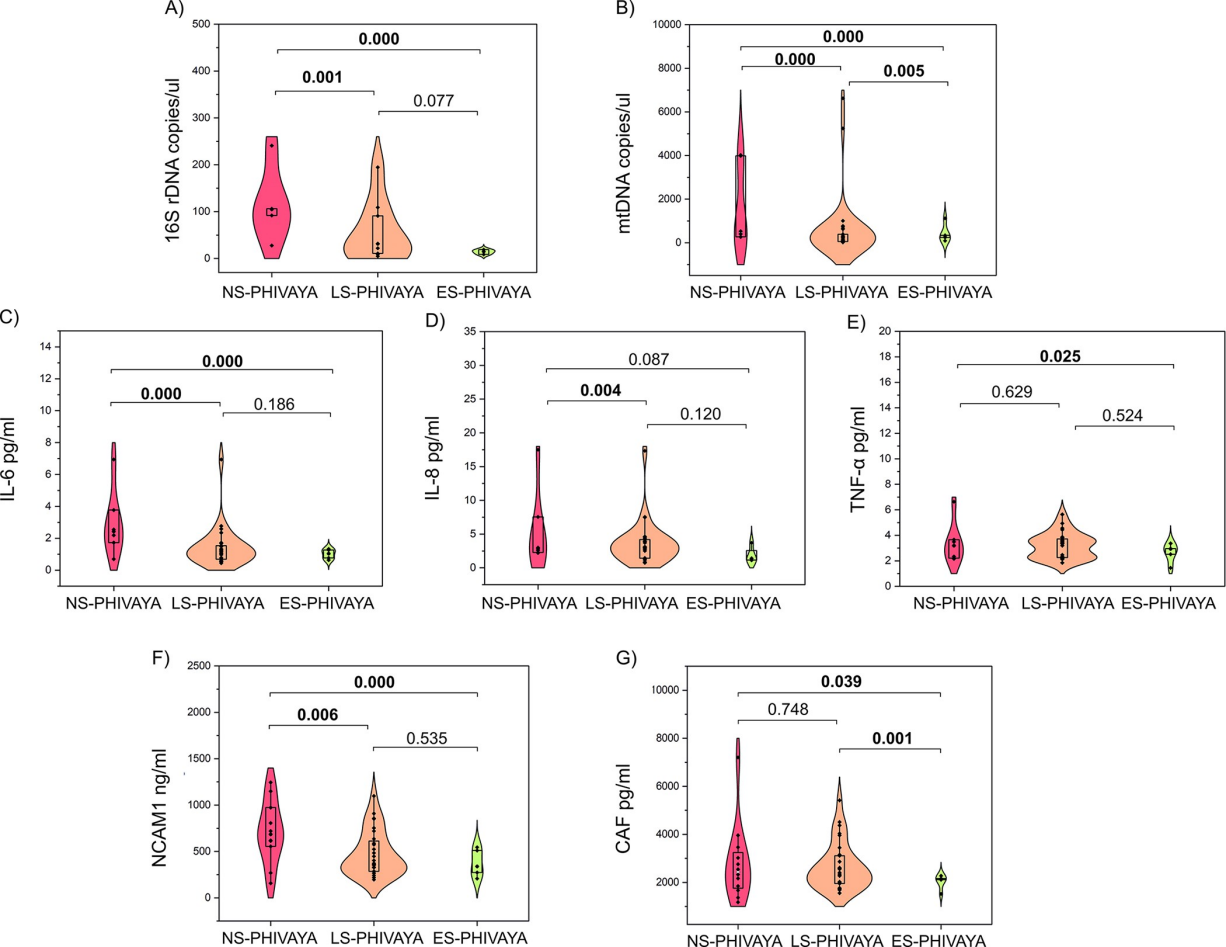
Comparison of circulating biomarkers among PHIVAYA subgroups. Circulating levels of 16S rDNA (PAMPs) (A), mtDNA (DAMPs) (B), and pro-inflammatory cytokines IL-6 (C), IL-8 (D) and TNF-α (E), and muscle wasting and denervation biomarkers, NCAM1 (F) and CAF (G) in PHIVAYA subgroups; p-values were adjusted for age, time on ART and time of ART initiation.

The persistence of the HIV reservoir may lead to the release of PAMPs and DAMPs into circulation, resulting in a chronic state of inflammation and immune activation [23]. Therefore, we analysed levels of PAMPs (as 16S rDNA), DAMPs (as mtDNA), and pro-inflammatory cytokines (IL-6, IL-8 and TNF-α) in PHIVAYA subgroups. ES-PHIVAYA had lowest circulating levels of PAMPs and pro-inflammatory cytokines than LS- and NS-PHIVAYA, while levels of mtDNA were significantly lower compared to NS-PHIVAYA, but higher than in LS-PHIVAYA (Fig 5 and S3 Table).

Notably, HIV-DNA positively correlated with circulating levels of PAMPs, DAMPs, IL-6, IL-8 and TNF-α, while no correlation was found with cell-associated HIV-RNA, except for IL-6 (Fig 4 and S2 Table).

Additional observations can be made from the correlation plot depicted in Fig 4: PAMPs tended to positively correlate with senescent CD4 (r = 0.71, p = 0.052) and CD8 cells (r = 0.60 and p = 0.077). Pro-inflammatory cytokines positively correlated with activated CD4 (TNF-α: r = 0.36, p = 0.098), activated CD8 cells (with IL-6: r = 0.41, p = 0.025; and IL-8: r = 0.39, p = 0.054), and exhausted CD4 cells (IL-6: r = 0.37, p = 0.040; IL-8, r = 0.34, p = 0.089; TNF-α: r = 0.50, p = 0.007) (Fig 4).

Regarding markers of muscle wasting and denervation, ES-PHIVAYA displayed the lowest circulating levels of NCAM1, and CAF compared to both LS- and NS-PHIVAYA (Fig 5 and S3 Table). HIV-DNA levels were positively correlated with circulating levels of both NCAM1 and CAF (Fig 4 and S2 Table).

Of note, a subgroup of 22 PHIVAYA who developed one or more comorbidities were compared to 33 PHIVAYA without comorbidities (Table 1). The results showed that circulating levels of PAMPs, TNF-α and CAF were significantly higher in PHIVAYA with comorbidities than those without (S4 Table). Notably, PHIVAYA with comorbidities showed circulating CAF levels, ranging from 1,743 to 7,205 pg/mL, which align with those found in sarcopenic volunteers and in patients with low muscle mass [24].

### 5. Comparison of multifaceted aging biomarkers among PHIVAYA subgroups and healthy controls

The entire cohort of PHIVAYA was compared to healthy controls (S5 Table). While the percentage of CD4 cells did not statistically differ (35.6 [28.2–38.9] *vs* 36.6 [34.7–40.2], p = 0.088), PHIVAYA had a significantly higher percentage of CD8 cells than healthy controls (31.7 [25.3–40.2] *vs* 21.1 [18.0–25.3], p = 0.000), and thus, CD4/CD8 ratio was significantly lower in PHIVAYA compared to healthy controls (1.0 [0.8–1.4] *vs* 1.8 [1.4–2.2], p = 0.000). PHIVAYA had significantly higher levels of activated, senescent, and exhausted CD4 and CD8 T cells, and lower TREC levels and shorter telomere length than healthy controls (S5 Table). Furthermore, PHIVAYA had significantly higher levels of circulating markers, including PAMPs, DAMPS, pro-inflammatory cytokines IL-6, IL-8 and TNF-α, NCAM1 and CAF than healthy controls (S5 Table).

**Fig 6:**
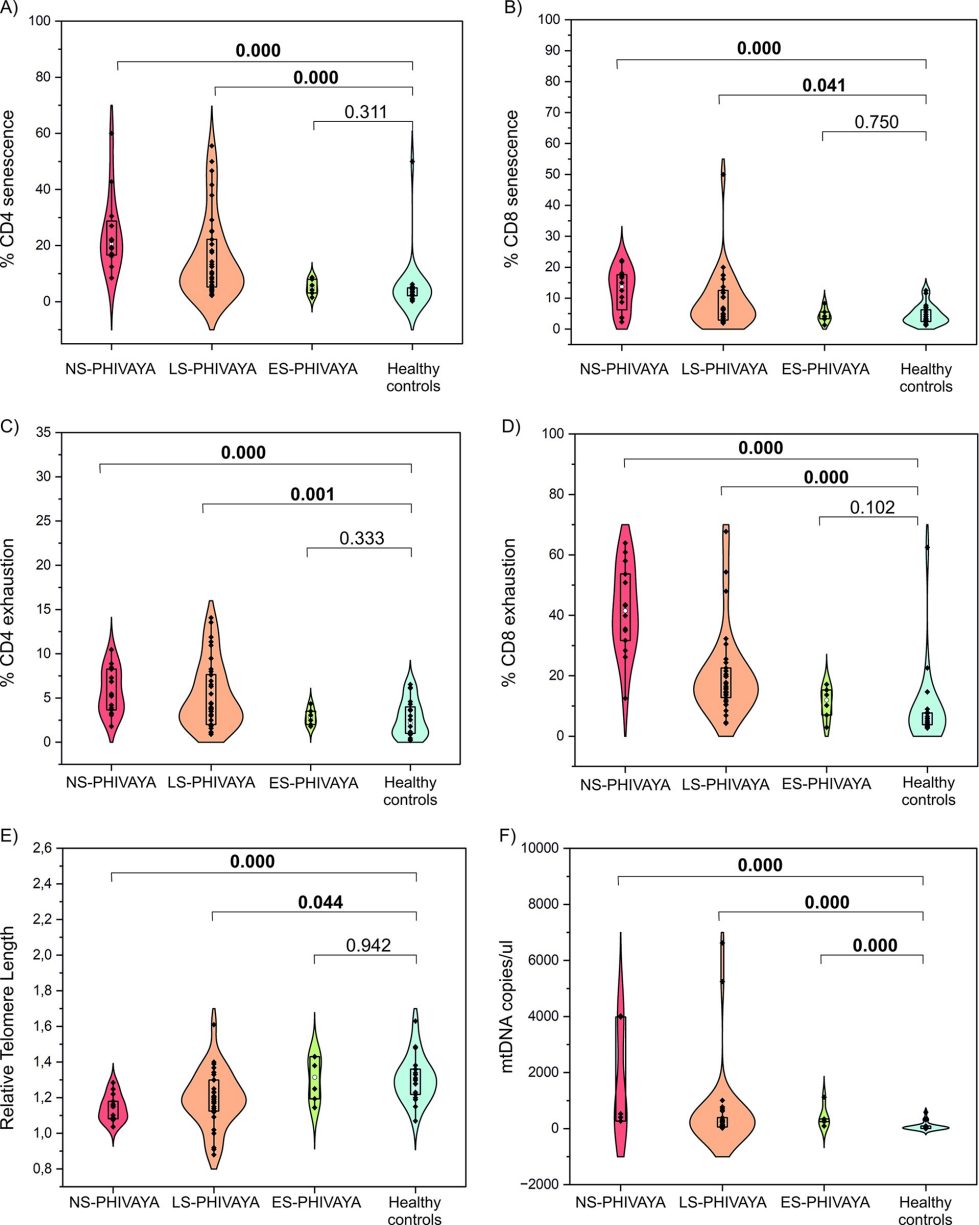
Comparison of markers of aging among PHIVAYA groups and healthy controls. Comparison of levels of immune senescent CD4 (A), CD8 (B), exhausted CD4 (C) and CD8 (D) cells, telomere length (E), and DAMPs (F) among PHIVAYA subgroups and healthy controls; p-values were adjusted for age.

When the PHIVAYA subgroups were individually compared to the healthy controls group, several important differences emerged (S5 Table). In particular, ES-PHIVAYA exhibited the lowest percentages of senescent and exhausted CD4 and CD8 cells than the other PHIVAYA subgroups, but similar to healthy controls (Fig 6 and S5 Table). Additionally, telomere lengths in ES-PHIVAYA were longer than those observed in the other PHIVAYA subgroups, and comparable to those of the healthy controls (Fig 6 and S5 Table). In addition, ES-PHIVAYA subgroup exhibited lower levels of PAMPs, pro-inflammatory cytokines IL-8 and TNF-α, NCAM1 and CAF than those observed in the other PHIVAYA subgroups, and comparable to those observed in healthy controls (S5 Table). Lastly, in ES-PHIVAYA circulating levels of DAMPS were lower than in NS-PHIVAYA, but significantly higher than healthy controls (Fig 6 and S5 Table).

## Discussion

Lifelong ART is essential for achieving sustained HIV suppression [25]. However, the persistence of the HIV reservoir can accelerate processes of immunosenescence and aging, ultimately leading to the onset of age-associated illnesses, including cancers, which represent a primary cause of death in the HIV population [4–7]. Remarkably, the United Nations Programme on HIV/AIDS (UNAIDS) estimated that by the end of 2022, there would be 1.5 million children (<15 years) living with HIV, with over 130,000 newly infected [26]; this represents a growing population at a higher risk of aging-associated diseases. In this study, for the first time, we have delineated a multifaceted aging profile in adolescents and young adults with perinatally acquired HIV, who have been infected with HIV since birth and are at heightened risk of aging-associated illnesses.

Despite over two decades of treatment, the latent reservoir remains a major barrier to cure HIV; it has been demonstrated that after an initial decay period spanning several years, the viral reservoir does not further decrease [27]. The persistence of the HIV reservoir is driven by clonal expansion of a few clones containing intact, replication-competent proviruses capable of producing infectious virions [20], but it primarily consists of defective proviruses. Recently, it has been demonstrated that defective, replication-incompetent proviruses may be transcriptionally active and capable of producing viral proteins, ultimately contributing to chronic inflammation and immune activation [21,22]. We found that the levels of unspliced HIV-RNA, which can represent the full-length genomic HIV-RNA, were significantly lower than total cell-associated HIV-RNA. Therefore, the entire size of the HIV reservoir was estimated according to the value of total proviral DNA and total cell-associated HIV-RNA. Findings that viral reservoir was significantly lower in ES-PHIVAYA indicate that early ART limits the size of the HIV reservoir; how the timing of therapy initiation influences the proportion of intact replication-competent proviruses *vs* defective proviruses remains an interesting question.

The persistence of HIV appears to be a key driver of premature senescence. HIV can induce the release of PAMPs and DAMPs into circulation, leading to chronic inflammation and persistent immune activation [23], thereby contributing to the onset of a senescent pathway/phenotype. Indeed, in agreement with previous findings in early treated PHIV adolescents [15,16,28], we found that HIV-DNA positively correlated with immune activated, exhausted and senescent T and B cells. Moreover, we also found that the increased levels of 16S rDNA, mtDNA, and pro-inflammatory cytokines correlated with immune activation, which, in turn, was associated with immune exhaustion and senescence. Importantly, even after adjusting for age, time on ART and time of ART initiation, the HIV reservoir correlated with markers of aging, thus supporting that HIV *itself* plays a role in inducing immune senescence and exhaustion. Furthermore, we found that PHIVAYA exhibit higher levels of immune activation, senescence and exhaustion compared to controls, in line with findings in PHIV children (aged 0–5 years) [14]. Fastenackels and colleagues [17] reported increased immune activation, immune senescence and inflammation (quantified as serum C-reactive protein levels) in young adults living with HIV compared with age-matched uninfected individuals; however, unlike our study population, in their research, these markers were associated with uncontrolled viral replication.

Regarding cellular senescence, levels of TREC were significantly lower in all PHIVAYA subgroups compared to age-matched healthy controls. This contrasts with the findings of two recent studies, which did not observe differences in TREC levels in young adults with perinatally acquired HIV compared to age-matched controls [18] or compared to young adults with non-perinatally acquired HIV [19]. Notably, NS-PHIVAYA had lower levels of TREC than S-PHIVAYA, suggesting that reduced TREC levels in blood may result not only from diminished thymic output, but also from a more rapid conversion into activated memory cells.

Telomere length is also significantly shorter in PHIVAYA than in healthy controls. This finding aligns with other studies [14,18]. Furthermore, we found an inverse correlation of telomere length with HIV-DNA, confirming that viral persistence, likely by inducing chronic immune activation and cell replication, impacts telomere length [15].

Of significant importance, the small group of ES-PHIVAYA, who initiated ART within the first year of life and achieved sustained viral suppression, exhibited lowest HIV reservoir size, lowest inflammatory profile and lowest percentages of senescent and exhausted T and B cells compared to the other PHIVAYA subgroups. Consistently, effective ART treatment initiated within 6 months of life in children living with PHIV dampens the long-term inflammatory plasma profile compared to late ART initiation [29]. Interestingly, ES-PHIVAYA exhibited levels of inflammatory markers, immune senescence and exhausted profile comparable to those of healthy controls. Notably, the ES-PHIVAYA had higher levels of TREC levels than the other PHIVAYA subgroups. This was consistent with previous studies [15,30], describing that delayed treatment in adolescents with perinatally acquired HIV correlates with lower TREC levels in PBMC. Additionally, telomere lengths in ES-PHIVAYA were longer than in other PHIVAYA subgroups and comparable to those of age-matched healthy controls, suggesting a low rate of immune activation and cellular replication in these subjects. These findings indicated that very early ART initiation by restricting the size of HIV reservoir, constrains the premature cellular aging.

T and B regulatory cells play a role in maintaining immune homeostasis by reducing systemic immune activation and promoting appropriate immune responses [31–35]. Their role in HIV infection is still controversial; they can either suppress generalized T-cell activation, which is beneficial, or impede protective anti-HIV cell-mediated immunity, contributing to viral persistence [35]. In our cohort, PHIVAYA had higher levels of T and B regulatory cells compared to controls, particularly in the subgroup of ES- PHIVAYA. The percentages of these cells were inversely correlated with levels of HIV-DNA and cell-associated HIV-RNA, and negatively correlated with immune activation, suggesting that regulatory cells by restricting immune activation, ultimately reduce the size of HIV reservoir and immune exhaustion and senescence.

However, it should be underlined that ES-PHIVAYA, although exhibiting a significantly better profile compared to LS- and NS-PHIVAYA, still display a residual detectable HIV reservoir and a low grade of inflammation. Notably, while levels of PAMPs were comparable to those of controls, their levels of DAMPs, although lower than those of the NS- and LS- PHIVAYA subgroups, remained significantly higher compared to controls, and they likely play a role in driving the low grade of inflammation and cellular activation in ES-PHIVAYA. The reason of this increased levels of DAMPs remains an open question.

Aging is associated with the progressive loss of muscle mass and function [24]. Recently, frailty, which includes measures of sarcopenia and muscle weakness, has been linked to mortality and comorbidity in subjects aged ≥45 years living with HIV [36]. CAF and NCAM1 have been identified as muscle wasting and neuromuscular junction denervation biomarkers [10,24]. In this study, for the first time, NCAM1 and CAF have been evaluated as functional markers of aging in PHIVAYA. Circulating levels of NCAM1 and CAF in the ES-PHIVAYA subgroup were similar to those of healthy controls, and significantly lower than the other NS- PHIVAYA and LS- PHIVAYA subgroups, and their levels were significantly correlated with the HIV reservoir. Of interest, PHIVAYA with comorbidities showed circulating CAF levels consistent with those found in sarcopenic volunteers and in patients with low muscle mass [24]. In addition, recent findings have reported an association between aging-related diseases, such as cardiac dysfunction, and inflammatory profile in children and young adults living with HIV [37]. Overall, the comorbidities observed in our cohort are not strictly defined as aging-associated diseases. Nonetheless, the increased immune activation and high levels of biomarkers, such as PAMPs, TNF-α and CAF found in PHIVAYA with comorbidities may indicate a vulnerable condition that could precede clinical manifestations. The ongoing follow-up of these patients will allow for the evaluation of the prognostic value of these markers.

**Fig 7:**
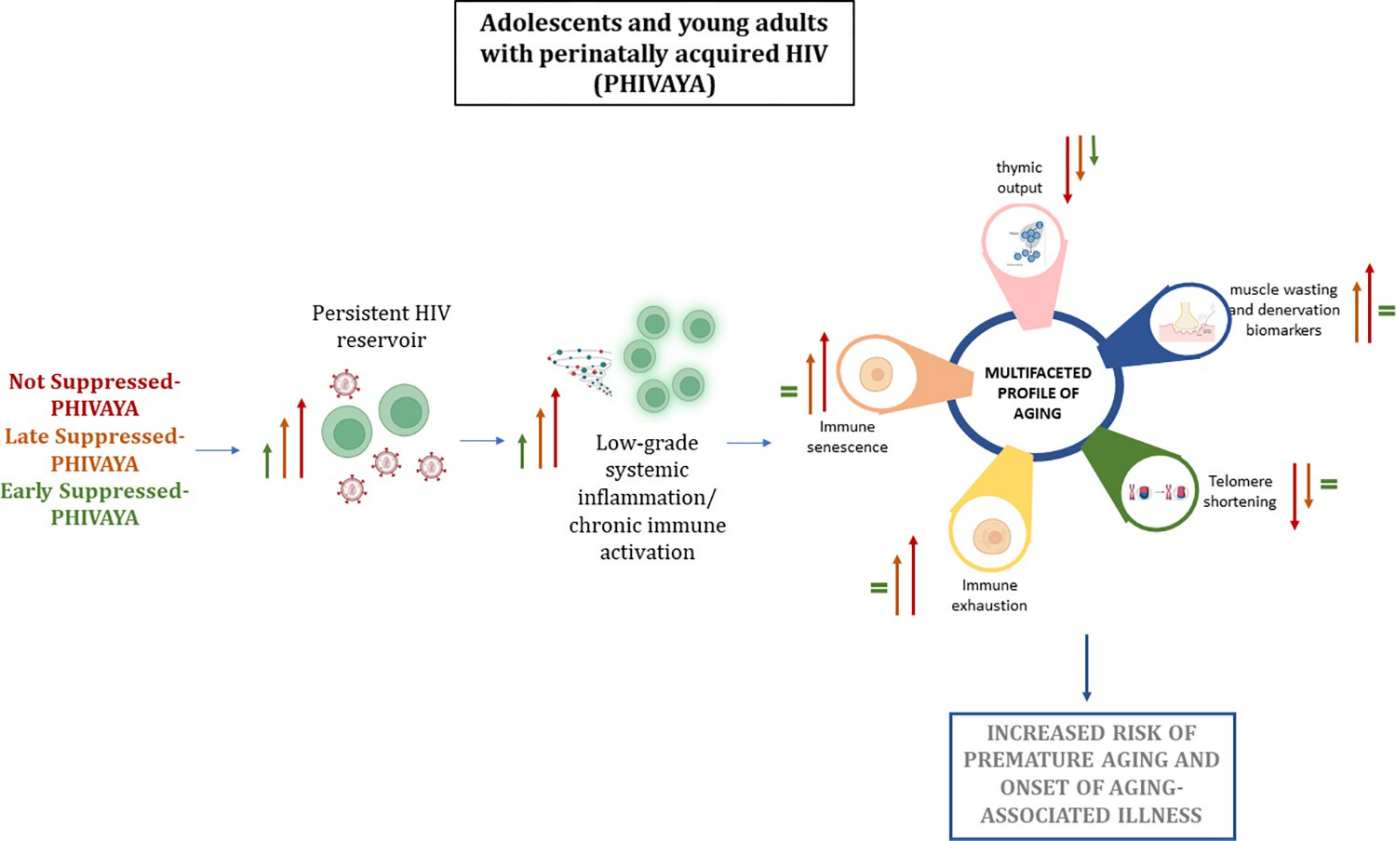
Schematic pathogenetic pathway of how persistent HIV reservoir may lead to premature aging and precocious onset of aging-related comorbidities. ART does not eradicate the virus, which persists as proviral HIV-DNA and residual cell-associated HIV-RNA. This persistence, likely through the damage of the gut mucosa and the release of PAMPs and DAMPs into the circulation, leads to a chronic inflammatory/immune activated condition, which in turn causes cellular and immunological senescence, including increased immune senescence and exhaustion, decreased thymic output, shorter telomeres, and higher levels of muscle wasting and denervation biomarkers. This multifaceted premature aging profile adds new tools for the minimally invasive monitoring of premature/accelerated aging-associated status in people at high risk since they have been living with the virus for their entire lives. The Not Suppressed (NS)-PHIVAYA subgroup displayed the highest (red arrow) HIV reservoir size, degree of inflammation (measured as levels of PAMPs, DAMPs, and proinflammatory cytokines), highest immune senescence and exhaustion, lowest levels of TREC, shorter telomere length and highest levels of NCAM1 and CAF compared to Late Suppressed (LS) (orange arrow) and Early Suppressed (ES) (green arrow) PHIVAYA.

In conclusion, while successful ART has extended the lifespan of people living with HIV, this population may experience premature aging, leading to the early onset of aging-associated illnesses, including cancer. Here, we have described a multifaceted aging profile (Fig 7), with biological, immunological, and functional biomarkers, as potential tools for non-invasive monitoring of the aging profile. Data provided from this study support the concept that early ART initiation and sustained viral suppression restrict the viral reservoir and constrain premature aging. Findings that LS-PHIVAYA exhibited lower levels of CD4 activation, senescence, and CD8 exhaustion, as well as reduced levels of PAMPs, DAMPs, proinflammatory cytokines IL-6 and IL-8, alongside higher levels of TREC and longer telomeres compared to NS-PHIVAYA, but significantly different from those of age-matched controls, support that the sustained viral suppression over time is a key determinant to limit premature senescence.

Moreover, findings that circulating markers of inflammation and denervation/sarcopenia correlate with cellular senescence open up new avenues for minimally invasive monitoring of adolescents and young adults with perinatally acquired HIV to assess their risk of age-related illnesses.

A limitation of this study is the small size of the three groups, especially the ES-PHIVAYA group. This constraint is understandable given the approach to ART 20 years ago. Nonetheless, aging in this population remains understudied, and even with small group sizes, our results contribute insights into this underexplored area.

It is emerging that early ART initiation may create conditions conducive to long-term post-treatment control and support the concept of analytic treatment interruption (ATI) as a strategy to achieve ART-free remission for infants with perinatally acquired HIV [38,39]. One open question is the parameters to select infants who can undergo to ATI [40,41]. Our results suggest that criteria for selecting candidates for treatment interruption may be based not only on viral load and CD4 count but also on a more comprehensive panel of markers, including chronic immune activation and aging biomarkers.

## Materials and methods

### Ethics statement

This study was approved by the Ethics Committee of Azienda Ospedaliera Padova (Approval number: #2921P) for the PHIVAYA study population, and by the Ethics Committee according to national regulations (Approval number: 71779) for the control group.

Study participants (aged 18 years and older), or the parents/legal guardians of those under 18, provided written informed consent in accordance with the Declaration of Helsinki.

### Study population and sampling

A total of 55 PHIVAYA, who attended the Department of Women’s and Children’s Health of the University Hospital of Padova, were included in this study. All study participants have been followed since birth and at the time of inclusion has been treated with ART. Plasmaviremia was recorded at diagnosis, ART initiation, throughout treatment and at the time of sampling. One subgroup of 41 participants, with undetectable HIV-RNA plasma levels (<50 copies/ml) and with no more than one annual viral blip of HIV-RNA (from 50 to 400 HIV-RNA copies/ml) for at least the last 10 years of follow-up has been identified as Suppressed PHIVAYA (S-PHIVAYA). The 14 remaining subjects, characterized by transient periods of detectable viremia (>1000 copies/ml), likely due to poor drug adherence and/or resistant mutations, were defined as Not Suppressed PHIVAYA (NS-PHIVAYA). Among the S-PHIVAYA, a small subgroup (n.6), characterized by undetectable HIV-RNA plasma levels (<50 copies/ml) achieved after viral suppression within 12 months of ART initiation and with no more than one annual viral blip during the time of follow-up, has been identified as Early Suppressed (ES-PHIVAYA), and the other 35 as Late Suppressed (LS-PHIVAYA). A control group of 23 gender- and age-matched healthy adolescents and young adults was included in the study (Fig 1).

Blood samples were collected in EDTA-containing tubes. Peripheral blood mononuclear cells (PBMC) and plasma were separated by centrifugation on a Ficoll-Paque gradient (Pharmacia, Uppsala, Sweden) and appropriately stored in liquid nitrogen and at -80°C, respectively, until use.

### DNA and RNA extraction

DNA was extracted from PBMC using QIAamp DNA Mini Kit (Qiagen, Hilden, Germany), as already described [14]. RNA from PBMC was extracted using Maxwell RSC simplyRNA Blood Kit on the Maxwell RSC 48 Instruments (Promega, Madison, WI, USA), following the manufacturer’s instruction. The concentration of eluted RNA was measured by Implen Nanophotometer 15920.

### HIV-DNA quantification by Droplet Digital PCR

HIV-DNA levels were measured in total PBMC by using the QX200 Droplet Digital PCR (ddPCR) system (Bio-Rad, Pleasanton, CA), as already reported [15]. The HIV copy number was normalized against the copy number of the TERT housekeeping gene, and the final results were expressed as HIV-DNA copies/10^6^ PBMC.

### Cell-associated HIV-RNA quantification by One-Step RT Droplet Digital PCR

The content of total and unspliced cell-associated HIV-RNA was quantified using in house quantitative assay based on One-Step RT ddPCR (Bio-Rad, Pleasanton, CA), with primers and probe previously reported [15,42]. In particular, we employed primers for the LTR region, that is present in all cell-associated HIV-RNA species, to measure the total cell-associated RNA, and primers for gag/pol region to measure the unspliced RNA, whose detection indicates the presence of full-length genomic HIV-RNA, able to produce infectious virions [42]. Levels of total and unspliced cell-associated HIV-RNA were assessed against the reference gene importin 8 (IPO8) (Bio-Rad, Pleasanton, CA), and expressed as total and unspliced HIV-RNA copies/10^6^ IPO8 in PBMC.

### Telomere length measurement

Relative telomere length (RTL) was determined in DNA extracted from PBMC by multiplex quantitative Real-time PCR, as previously described [14, 15]. All DNA samples and reference samples were run in triplicate. RTL values were calculated as telomere/single-copy gene ratio, as previously described [14].

### Thymic output quantification

Thymic output in PBMC was studied by measurement of TREC levels by real-time PCR, as previously described [43]. TREC levels were expressed as TREC copies/10^6^ PBMC.

### Flow cytometry

Whole blood was routinely stained with BD Multitest CD3/CD8/CD45/CD4 (Becton-Dickinson, San Diego, CA, USA) to obtain total frequencies of CD4 and CD8 cells and calculate the ratio CD4/CD8. The specific profile of activated, senescent, exhausted and regulatory T and B cells was studied by flow cytometry. Cells were thawed, washed, and stained for 20 min in the dark with the Live/Dead Fixable Near-IR Dead Cell Stain Kit (Life Technologies, Carlsbad, California, USA) and the following labelled monoclonal antibodies: anti-CD3 [FITC], anti-CD4 [PerCP], anti-CD38 [phycoerythrin (PE)], anti-HLA-DR [allophycocyanin (APC)], anti-CD27 [PE], anti-CD45RA [APC], anti-CCR7 [PE-Cy7], anti-CD28 [BV421], anti-CD57 [PE-CF594], anti-TIGIT [BV605], anti-CD21 [BV421], anti-CD27 [PE-Cy7], anti-IgD [PE] (Becton-Dickinson-BD, San Diego, California, USA); anti-CD8 [VioGreen], anti-CD19 [VioBright515], anti-CD10 [APC] (Miltenyi Biotec, Auburn, California USA). The cells were then washed and resuspended in PBS supplemented with 1% paraformaldehyde. T-regs were determined using anti-CD4 [BB515], anti-CD25 [BV421], anti-CD127 [PE-CF594] (Becton-Dickinson, San Diego, CA, USA) and combined membrane and intracytoplasmic staining for anti-FoxP3 [AlexaFluor 647] using a Transcription Factor Buffer Set according to the manufacturer’s protocol (Becton-Dickinson, San Diego, CA, USA).

All samples were analysed using an LSRII Flow cytometer (Becton-Dickinson). A total of 50000 events were collected in the lymphocyte gate using morphological parameters (forward and side-scatter). Data were processed with FACSDiva Software (Becton-Dickinson) and analysed using Kaluza Analyzing Software v.1.2 (Beckman Coulter, Brea, CA, USA) (S1 Fig).

### Circulating levels of PAMPs, DAMPs and pro-inflammatory cytokines

DNA was extracted from 200 μl of plasma using the QIAamp DNA Mini Kit (QIAGEN, Hilden, Germany) and eluted in 50 μl of AE buffer. To estimate circulating levels of 16S ribosomal (r)DNA, 5 μl of DNA were amplified by Real-Time PCR, using primers and probe as already reported [44]. Results were expressed as 16S rDNA copies/μl of plasma.

To quantify circulating levels of mitochondrial (mt) DNA, a quantitative method based on real-time PCR assay was performed with primers pair and probe as previously described [44]. Results were expressed as mtDNA copies/μl of plasma.

Circulating levels of pro-inflammatory cytokines were quantified using a Luminex platform (Human Cytokine Discovery, R&D System, Minneapolis, MN) for the simultaneous detection of the following molecules: IL-6, IL-8, IL-17, TNF-α, according to the manufacturer’s instruction. Results were expressed as pg/ml of plasma.

### Circulating levels of C-terminal Agrin Fragment (CAF) and Neural Cell Adhesion Molecule 1(NCAM1)

Plasma levels of CAF and NCAM1 were determined using commercially available enzyme-linked immunosorbent assay (ELISA) kits (ab216945 Human Agrin SimpleStep, Abcam, Cambridge, UK, and RayBio Human NCAM1 ELISA Kit, RayBiotech Norcross, Georgia, USA, respectively), following the manufacturer’s instructions. All samples were run in duplicate. Plasma samples were diluted 1:10 using the appropriate diluent purchased with the kit. Concentrations of CAF and NCAM1 were read at 450 nm at Victor X3 (PerkinElmer, Waltham, MA, USA), interpolated from the respective standard curve, corrected for sample dilution, and expressed as pg/ml and ng/ml, respectively.

### Statistical analyses

Continuous variables were summarized using median and interquartile range (IQR) and their distributions among PHIVAYA subgroups were compared using the Kruskal-Wallis test adjusted for age. The correlation between variables was investigated through the Spearman rank correlation and the partial Spearman rank correlation was adjusted for age, time on ART and time of ART initiation. All statistical tests were two-sided and a p-value <0.05 was considered statistically significant. Statistical analyses were performed using the RStudio (RStudio: Integrated Development for R. RStudio Inc., Boston, MA, US).

## Supporting information

S1 Table (A) Comparison of HIV-DNA and cell-associated HIV-RNA levels between Not Suppressed (NS)- and Suppressed (S)-PHIVAYA subgroups. (B) Comparison of HIV-DNA and cell-associated HIV-RNA levels among all PHIVAYA subgroups. (DOCX)

S2 Table Correlation between HIV-DNA and cell-associated HIV-RNA and multifaceted studied parameters in all PHIVAYA. (DOCX)

S3 Table Comparison of circulating biomarkers among Not Suppressed (NS)-, Late Suppressed (LS)- and Early Suppressed (ES)-PHIVAYA. (DOCX)

S4 Table Comparison of multifaceted aging biomarkers between PHIVAYA with comorbidities and PHIVAYA without comorbidities. (DOCX)

S5 Table Comparison of multifaceted aging biomarkers between all PHIVAYA and each PHIVAYA subgroups with healthy controls. (DOCX)

S1 Fig Flow cytometry gating strategy for (A) total lymphocytes on morphological parameters forward scatter (FSC) and side scatter (SSC); within the lymphocyte gate, selection on FSC-height (H) versus FSC-area (A) to exclude doublet cells; to identify only live cells, a negative selection for live/dead markers. (B) The strategy to gate on CD3+CD4+ and CD3+CD8+ T cell (C) senescence (CD28-CD57+), (D) activation (HLA-DR+CD38+), (E) exhaustion (PD-1+ and TIGIT+); and (G) Tregs (CD4+CD25+CD127-FoxP3+). (F) Flow cytometry gating strategy for B cell activation (CD19+CD10-CD27+CD21-), senescence (CD19+IgD-CD27-) and Bregs (CD19+CD24hiCD38hi). (TIF)

## References

[1] Deeks SG. HIV infection, inflammation, immunosenescence, and aging Annu Rev Med. 2011. 62:141–55. doi: 10.1146/annurev-med-042909-093756 21090961 PMC3759035

[2] Pitman MC, Lau JSY, McMahon JH, Lewin SR. Barriers and strategies to achieve a cure for HIV. Lancet HIV. 2018; 5(6): e317–e328. doi: 10.1016/S2352-3018(18)30039-0 29893245 PMC6559798

[3] Rodés B, Cadiñanos J, Esteban-Cantos A, Arribas JR. Ageing with HIV: Challenges and biomarkers. EBioMedicine. 2022: 77: 103896. doi: 10.1016/j.ebiom.2022.103896 35228014 PMC8889090

[4] Chiappini E, Bianconi M, Dalzini A, Petrara MR, Galli L, Giaquinto C, et al. Accelerated aging in perinatally HIV-infected children: clinical manifestations and pathogenetic mechanisms. Aging (Albany NY). 2018;10(11):3610–3625. doi: 10.18632/aging.101622 30418933 PMC6286860

[5] Dalzini A, Petrara MR, Ballin G, Zanchetta M, Giaquinto C, De Rossi A. Biological Aging and Immune Senescence in Children with Perinatally Acquired HIV. J Immunol Res. 2020; 2020: 8041616. doi: 10.1155/2020/8041616 32509884 PMC7246406

[6] Gooden TE et al. A matched cohort study investigating premature, accentuated, and accelerated aging in people living with HIV. HIV Med. 2023 May;24(5):640–647. doi: 10.1111/hiv.13375 35934808

[7] Horner MJ, Shiels MS, Pfeiffer RM, Engels ES. Deaths Attributable to Cancer in the US Human Immunodeficiency Virus Population During 2001–2015. Clin Infect Dis. 2021;72(9):e224–e231. doi: 10.1093/cid/ciaa1016 32710777 PMC8096269

[8] Power GA, Dalton BH, Rice CL. Human neuromuscular structure and function in old age: A brief review. J Sport Health Sci. 2013; 2(4): 215–226. doi: 10.1016/j.jshs.2013.07.001 27011872 PMC4801513

[9] Niezgoda A, Michalak S, Losy J, Kalinowska-Łyszczarz A, Kozubski W. sNCAM as a specific marker of peripheral demyelination. Immunol Lett. 2017: 185: 93–97. doi: 10.1016/j.imlet.2017.03.011 28336415

[10] Landi F, Calvani R, Lorenzi M, Martone AM, Tosato M, Drey M, et al. Serum levels of C-terminal agrin fragment (CAF) are associated with sarcopenia in older multimorbid community-dwellers: Results from the ilSIRENTE study. Exp Gerontol. 2016; 79: 31–36. doi: 10.1016/j.exger.2016.03.012 27015736

[11] Alvarez S, Brañas F, Sánchez-Conde M, Moreno S, López-Bernaldo de Quirós JC, Muñoz-Fernández MÁ. Frailty, markers of immune activation and oxidative stress in HIV infected elderly. PLoS One. 2020;15(3): e0230339. doi: 10.1371/journal.pone.0230339 32187205 PMC7080240

[12] Erlandson KM J, Allshouse AA, Jankowski CM, Lee EJ, Rufner KM, Palmer BE, et al. Association of functional impairment with inflammation and immune activation in HIV type 1-infected adults receiving effective antiretroviral therapy. Infect Dis. 2013; 208: 249–59. doi: 10.1093/infdis/jit147 23559466 PMC3685225

[13] Margolick JB, Bream JH, Martínez-Maza O, Lopez J, Li X, Phair JP, et al. Frailty and Circulating Markers of Inflammation in HIV+ and HIV- Men in the Multicenter AIDS Cohort Study. J Acquir Immune Defic Syndr. 2017; 74: 407–417. doi: 10.1097/QAI.0000000000001261 28225718 PMC5365031

[14] Gianesin K, Noguera-Julian A, Zanchetta M, Del Bianco P, Petrara MR, Freguja R, et al. Premature aging and immune senescence in HIV-infected children. AIDS. 2016; 30(9): 1363–73. doi: 10.1097/QAD.0000000000001093 26990630 PMC4867984

[15] Dalzini A, Ballin G, Dominguez-Rodriguez S, Rojo P, Petrara MR, Foster C, et al; EPIICAL Consortium. Size of HIV-1 reservoir is associated with telomere shortening and immunosenescence in early-treated European children with perinatally acquired HIV-1. J Int AIDS Soc. 2021; 24(11): e25847. doi: 10.1002/jia2.25847 34797948 PMC8604380

[16] Rinaldi S, de Armas L, Dominguez-Rodríguez S, Pallikkuth S, Dinh V, Pan L, et al; EPIICAL consortium. T cell immune discriminants of HIV reservoir size in a pediatric cohort of perinatally infected individuals PLoS Pathog. 2021; 17(4):e1009533. doi: 10.1371/journal.ppat.1009533 33901266 PMC8112655

[17] Fastenackels S, Sauce D, Vigouroux C, Avettand-Fènoël V, Bastard JP, Fellahi S, et al; ANRS Co19 COVERTE Study Group. HIV-mediated immune aging in young adults infected perinatally or during childhood. AIDS. 2019; 33(11):1705–1710. doi: 10.1097/QAD.0000000000002275 31149945

[18] Paghera S, Quiros-Roldan E, Sottini A, Properzi M, Castelli F, Imberti L. Lymphocyte homeostasis is maintained in perinatally HIV-infected patients after three decades of life. Immun Ageing. 2019:16:26. doi: 10.1186/s12979-019-0166-7 31636688 PMC6791008

[19] Quiros-Roldan E, Properzi M, Paghera S, Raffetti E, Castelli F, Imberti L. Factors associated with immunosenescence during early adulthood in HIV-infected patients after durable efficient combination antiretroviral therapy. Sci Rep. 2020; 10(1):10057. doi: 10.1038/s41598-020-67100-8 32572110 PMC7308364

[20] Simonetti FR, Sobolewski MD, Fyne E, Shao W, Spindler J, Hattori J, et al. Clonally expanded CD4+ T cells can produce infectious HIV-1 in vivo. Proc Natl Acad Sci USA 2016; 113:1883–1888. doi: 10.1073/pnas.1522675113 26858442 PMC4763755

[21] Imamichi H, Smith M, Adelsberger JW, Izumi T, Scrimieri F, Sherman BT, et al. Defective HIV-1 proviruses produce viral proteins. Proc Natl Acad Sci U S A. 2020 Feb 18;117(7):3704–3710. doi: 10.1073/pnas.1917876117 32029589 PMC7035625

[22] Singh K, Natarajan V, Dewar R, Rupert A, Badralmaa Y, Zhai T, et al. Long-term persistence of transcriptionally active ’defective’ HIV-1 proviruses: implications for persistent immune activation during antiretroviral therapy. AIDS. 2023 Nov 15;37(14):2119–2130. doi: 10.1097/QAD.0000000000003667 37555786 PMC10615727

[23] Douek DC. Immune Activation, HIV Persistence, and the Cure. Top Antivir Med. 2013; 21(4): 128–132. . 24225078 PMC6148844

[24] Monti E, Sarto F, Sartori R, Zanchettin G, Löfler S, Kern H, et al. C-terminal agrin fragment as a biomarker of muscle wasting and weakness: a narrative review. J Cachexia Sarcopenia Muscle. 2023; 14(2): 730–744. doi: 10.1002/jcsm.13189 36772862 PMC10067498

[25] Wandeler G, Johnson LF, Egger M. Trends in life expectancy of HIV-positive adults on antiretroviral therapy across the globe: comparisons with general population Curr Opin HIV AIDS. 2016;11(5):492–500. doi: 10.1097/COH.0000000000000298 27254748 PMC5055447

[26] UNAIDS. UNAIDS Global AIDS Update 2023. March 22, 2024; https://www.unaids.org/en/resources/documents/2023/2023_unaids_data.

[27] McMyn NF, Varriale J, Fray EJ, Zitzmann C, MacLeod H, Lai J, et al. The latent reservoir of inducible, infectious HIV-1 does not decrease despite decades of antiretroviral therapy. J Clin Invest. 2023 Sep 1;133(17):e171554. doi: 10.1172/JCI171554 37463049 PMC10471168

[28] Huang Y, Dhummakupt A, Khetan P, Nilles T, Zhou W, Mudvari P, et al. Immune activation and exhaustion marker expression on T-cell subsets in ART-treated adolescents and young adults with perinatal HIV-1 infection as correlates of viral persistence. Front Immunol. 2023; 14: 1007626. doi: 10.3389/fimmu.2023.1007626 37033916 PMC10076634

[29] Nguyen AN, Plotkin AL, Odumade OA, De Armas L, Pahwa S, Morrocchi E, et al; on the behalf of the EPIICAL Consortium. Effective early antiretroviral therapy in perinatal-HIV infection reduces subsequent plasma inflammatory profile Pediatr Res. 2023 Nov;94(5):1667–1674. doi: 10.1038/s41390-023-02669-0 37308683

[30] Domınguez-Rodrıguez S, Tagarro A, Foster C, Palma P, Cotugno N, Zicari S, Ruggiero A, de Rossi A, Dalzini A, Pahwa S, Rinaldi S, Nastouli E, Marcelin A-G, Dorgham K, Sauce D, Gartner K, Rossi P, Giaquinto C and Rojo P (2022) Clinical, Virological and Immunological Subphenotypes in a Cohort of Early Treated HIV-Infected Children. Front. Immunol. 13:875692. doi: 10.3389/fimmu.2022.875692 35592310 PMC9111748

[31] López-Abente J, Correa-Rocha R, Pion M. Functional Mechanisms of Treg in the Context of HIV Infection and the Janus Face of Immune Suppression. Front Immunol. 2016: 7: 192. doi: 10.3389/fimmu.2016.00192 27242797 PMC4871867

[32] Catalan D, Mansilla MA, Ferrier A, Soto L, Oleinika K, Aguillón JC, et al. Immunosuppressive Mechanisms of Regulatory B Cells. Front Immunol. 2021: 12: 611795. doi: 10.3389/fimmu.2021.611795 33995344 PMC8118522

[33] Schulze Zur Wiesch J, Thomssen A, Hartjen P, Tóth I, Lehmann C, Meyer-Olson D, et al. Comprehensive analysis of frequency and phenotype of T regulatory cells in HIV infection: CD39 expression of FoxP3+ T regulatory cells correlates with progressive disease J Virol.; 2011; 85(3):1287–97. doi: 10.1128/JVI.01758-10 21047964 PMC3020516

[34] Angin M, Kwon DS, Streeck H, Wen F, King M, Rezai A, et al. Preserved function of regulatory T cells in chronic HIV-1 infection despite decreased numbers in blood and tissue. J Infect Dis. 2012; 205(10):1495–500. doi: 10.1093/infdis/jis236 22427677 PMC3415814

[35] Chevalier MF, Weiss L. The split personality of regulatory T cells in HIV infection. Blood. 2013; 121(1): 29–37. doi: 10.1182/blood-2012-07-409755 23043072

[36] Verheij E, Kirk GD, Wit FW, van Zoest RA, Verboeket SO, Lemkes BA, et al; AGEhIV Cohort. Frailty Is Associated With Mortality and Incident Comorbidity Among Middle-Aged Human Immunodeficiency Virus (HIV)-Positive and HIV-Negative Participants. J Infect Dis. 2020; 222(6):919–928. doi: 10.1093/infdis/jiaa010 31956893 PMC7430168

[37] McCrary AW, Nyandiko WM, Ellis AM, Chakraborty H, Muehlbauer MJ, Koech MM, et al. Early cardiac dysfunction in children and young adults with perinatally acquired HIV. AIDS. 2020 Mar 15;34(4):539–548. doi: 10.1097/QAD.0000000000002445 31794518 PMC7050370

[38] Persaud D, Bryson Y, Nelson BS, Tierney C, Cotton MF, Coletti A, et al. HIV-1 reservoir size after neonatal antiretroviral therapy and the potential to evaluate antiretroviral-therapy-free remission (IMPAACT P1115): a phase 1/2 proof-of-concept study. Lancet HIV. 2024; 11: e20–e30. doi: 10.1016/S2352-3018(23)00236-9 38061376 PMC11094801

[39] Bengu N, Cromhout G, Adland E, Govender K, Herbert N, Lim N, et al. Sustained aviremia despite anti-retroviral therapy non-adherence in male children after in utero HIV transmission. Nat Med. 2024. doi: 10.1038/s41591-024-03105-4 38843818 PMC11485204

[40] Bekka S, Kelly K, Haaren M, Dhummakupt A, Persaud D. Age at ART initiation and proviral reservoir size in perinatal HIV-1 infection: considerations for ART-free remission. Curr Opin HIV AIDS. 2024;19(2):79–86. doi: 10.1097/COH.0000000000000839 38169427 PMC11715321

[41] Kuhn L, Barnabas S, Cotugno N, Peay H, Goulder P, Cotton M, et al; EPIICAL consortium. Analytical treatment interruption in children living with HIV: position statement from the EPIICAL consortium. Lancet HIV. 2024: S2352–3018(24)00157-7. doi: 10.1016/S2352-3018(24)00157-7 39059402 PMC12971027

[42] Gärtner K, Domínguez-Rodríguez S, Heaney J, Gkouleli T, Grant P, Dorgham K, et al. Low unspliced cell-associated HIV RNA in early treated adolescents living with HIV on long suppressive ART. Front Immunol. 2024; 15: 1334236. doi: 10.3389/fimmu.2024.1334236 38444847 PMC10912947

[43] Ometto L, De Forni D, Patiri F, Trouplin V, Mammano F, Giacomet V, et al. Immune reconstitution in HIV-1-infected children on antiretroviral therapy: role of thymic output and viral fitness. AIDS. 2002; 16(6):839–49. doi: 10.1097/00002030-200204120-00003 11919485

[44] Petrara MR, Bonfante F, Costenaro P, Cantarutti A, Carmona F, Ruffoni E, et al. Asymptomatic and Mild SARS-CoV-2 Infections Elicit Lower Immune Activation and Higher Specific Neutralizing Antibodies in Children Than in Adults. Front Immunol. 2021; 12: 741796. doi: 10.3389/fimmu.2021.741796 34659235 PMC8515185

