# Supplementary material for: HIV reservoir and premature aging: risk factors for aging-associated illnesses in adolescents and young adults with perinatally acquired HIV
Source: PLoS Pathog. 2024 Sep 23;20(9):e1012547. doi: 10.1371/journal.ppat.1012547 (PMC11449303; doi:10.1371/journal.ppat.1012547)
Supplement: S1 Table — (DOCX) [file ppat.1012547.s001.docx]

**Table A. Comparison of HIV-DNA and cell-associated HIV-RNA levels between Not Suppressed (NS)- and Suppressed (S)-PHIVAYA subgroups**

| **Parameters**  **Median [IQR]** | **NS-PHIVAYA**  **(N=14)** | **S-PHIVAYA**  **(N=41)** | **p-value*** | **p-value**** |
| --- | --- | --- | --- | --- |
| HIV-DNA copies/10^6^ PBMC | 208 [84-281] | 59 [31-142] | **0.003** | 0.160 |
| HIV-RNA copies/10^6^ IPO8 in PBMC | 704 [306-1432] | 354 [123-639] | **0.004** | 0.605 |
| unspliced HIV-RNA copies/10^6^ IPO8 in PBMC | 90 [26-158] | 18 [1-67] | **0.007** | 0.399 |

**adjusted by age*

*** adjusted by age, time on ART and time of ART initiation.*

**Table B. Comparison of HIV-DNA and cell-associated HIV-RNA levels among all PHIVAYA subgroups**

| **Parameters**  **Median [IQR]** | **(A)**  **NS-PHIVAYA**  **(N=14)** | **(B)**  **LS-PHIVAYA**  **(N=35)** | **(C)**  **ES-PHIVAYA**  **(N=6)** | **p-value**  **A vs B** | **p-value**  **A vs C** | **p-value**  **B vs C** |
| --- | --- | --- | --- | --- | --- | --- |
| HIV-DNA copies/10^6^ PBMC | 208 [84-281] | 70 [37-168] | 32 [27-47] | **0.023*** | **0.000*** | **0.023*** |
|  |  |  |  | 0.170** | **0.001**** | **0.002**** |
| HIV-RNA copies/10^6^ IPO8 in PBMC | 704 [306-1432] | 424 [126-675] | 118 [77-304] | 0.078* | **0.012*** | 0.465* |
|  |  |  |  | 0.796** | **0.018**** | 0.104** |
| unspliced HIV-RNA copies/10^6^ IPO8 in PBMC | 90 [26-158] | 26 [1-76] | 1 [1-30] | **0.048*** | **0.000*** | **0.005*** |
|  |  |  |  | 0.483** | **0.000**** | **0.002**** |

**adjusted by age*

*** adjusted by age, time on ART and time of ART initiation.*
