# Supplementary material for: HIV reservoir and premature aging: risk factors for aging-associated illnesses in adolescents and young adults with perinatally acquired HIV
Source: PLoS Pathog. 2024 Sep 23;20(9):e1012547. doi: 10.1371/journal.ppat.1012547 (PMC11449303; doi:10.1371/journal.ppat.1012547)
Supplement: S2 Table — (DOCX) [file ppat.1012547.s002.docx]

**S2 Table. Correlation between HIV-DNA and cell-associated HIV-RNA and multifaceted studied parameters in all PHIVAYA**Inizio modulo

| **HIV reservoir** | **Immunological markers** | **r* (95%CI)** | **p-value*** | **r** (95%CI)** | **p-value**** |
| --- | --- | --- | --- | --- | --- |
| HIV-DNA copies/10^6^ PBMC | % CD4+ activation | 0.40 (0.10;0.63) | **0.010** | 0.41 (0.12;0.64) | **0.008** |
|  | % CD8+ activation | 0.39 (0.12;0.61) | **0.006** | 0.39 (0.09;0.63) | **0.011** |
|  | % B activation | 0.53 (0.27;0.71) | **0.000** | 0.55 (0.28;0.73) | **0.000** |
|  | % CD4+ senescence | 0.40 (0.11;0.63) | **0.008** | 0.42 (0.14;0.65) | **0.005** |
|  | % CD8+ senescence | 0.40 (0.13;0.62) | **0.005** | 0.32 (0.03;0.55) | **0.028** |
|  | % B senescence | 0.36 (0.07;0.60) | **0.015** | 0.38 (0.07;0.63) | **0.019** |
|  | % CD4+ exhaustion | 0.61 (0.38;0.76) | **<0.000** | 0.59 (0.34;0.76) | **<0.000** |
|  | % CD8+ exhaustion | 0.71 (0.55;0.82) | **<0.000** | 0.73 (0.59;0.83) | **<0.000** |
|  | % T-regs | -0.61 (-0.77;-0.38) | **<0.000** | -0.64 (-0.81;-0.38) | **<0.000** |
|  | % B-regs | -0.41 (-0.62;-0.16) | **0.002** | -0.40 (-0.62;-0.13) | **0.005** |
|  | TREC | -0.20 (-0.49;0.12) | 0.210 | -0.24 (-0.51;0.07) | 0.133 |
|  | RTL | -0.32 (-0.58;0.01) | **0.050** | -0.27 (-0.56;0.07) | 0.056 |
|  | 16S rDNA copies/µl | 0.53 (0.07;0.80) | **0.027** | 0.61 (0.07;0.87) | **0.029** |
|  | mtDNA copies/µl | 0.36 (0.02;0.63) | **0.039** | 0.34 (-0.06;0.64) | 0.057 |
|  | IL-6 pg/ml | 0.50 (0.16;0.73) | **0.005** | 0.51 (0.18;0.74) | **0.004** |
|  | IL-8 pg/ml | 0.30 (-0.06;0.60) | 0.099 | 0.35 (0.0;0.63) | **0.050** |
|  | TNF-α pg/ml | 0.27 (-0.09;0.56) | 0.136 | 0.32 (-0.02;0.60) | **0.043** |
|  | NCAM1 ng/ml | 0.54 (0.24;0.74) | **0.001** | 0.53 (0.21;0.74) | **0.002** |
|  | CAF pg/ml | 0.34 (0.02;0.59) | **0.036** | 0.26 (-0.10;0.56) | 0.059 |
| HIV-RNA copies/10^6^ IPO8 in PBMC | % CD4+ activation | 0.26 (-0.03;0.50) | 0*.*080 | 0.22 (-0.08;0.48) | 0.149 |
|  | % CD8+ activation | 0.30 (-0.03;0.57) | 0.076 | 0.31 (-0.03;0.59) | 0.080 |
|  | % B activation | 0.28 (-0.04;0.55) | 0.090 | 0.33 (0.0;0.60) | 0.052 |
|  | % CD4+ senescence | 0.18 (-0.11;0.45) | 0.228 | 0.22 (-0.07;0.48) | 0.142 |
|  | % CD8+ senescence | 0.34 (0.00;0.61) | **0.050** | 0.28 (-0.06;0.56) | 0.103 |
|  | % B senescence | 0.06 (-0.23;0.35) | 0.677 | 0.11 (-0.20;0.41) | 0.489 |
|  | % CD4+ exhaustion | 0.11 (-0.18;0.38) | 0.446 | 0.11 (-0.18;0.39) | 0.456 |
|  | % CD8+ exhaustion | 0.33 (0.04;0.57) | **0.026** | 0.29 (-0.02;0.55) | 0.069 |
|  | % T-regs | -0.50 (-0.67;-0.25) | **0.000** | -0.51 (-0.69;-0.25) | **0.000** |
|  | % B-regs | -0.20 (-0.47;0.09) | 0.180 | -0.27 (-0.54;0.05) | 0.095 |
|  | TREC | 0.09 (-0.22;0.38) | 0.570 | 0.09 (-0.21;0.38) | 0.549 |
|  | RTL | -0.10 (-0.41;0.24) | 0.582 | -0.20 (-0.49;0.13) | 0.235 |
|  | 16S rDNA copies/µl | 0.13 (-0.45;0.63) | 0.681 | 0.19 (-0.46;0.71) | 0.583 |
|  | mtDNA copies/µl | 0.20 (-0.18;0.54) | 0.302 | 0.28 (-0.19;0.65) | 0.240 |
|  | IL-6 pg/ml | 0.36 (0.02;0.63) | **0.040** | 0.39 (0.03;0.67) | **0.036** |
|  | IL-8 pg/ml | 0.20 (-0.20;0.55) | 0.319 | 0.28 (-0.15;0.62) | 0.204 |
|  | TNF-α pg/ml | 0.28 (-0.08;0.57) | 0.125 | 0.25 (-0.11;0.55) | 0.174 |
|  | NCAM1 ng/ml | 0.19 (-0.18;0.52) | 0.314 | 0.20 (-0.18;0.52) | 0.303 |
|  | CAF pg/ml | 0.20 (-0.15;0.50) | 0.267 | 0.24 (-0.11;0.54) | 0.173 |

** Adjusted by age*

*** Adjusted by age, time on ART and time of ART initiation.*
