# Supplementary material for: HIV reservoir and premature aging: risk factors for aging-associated illnesses in adolescents and young adults with perinatally acquired HIV
Source: PLoS Pathog. 2024 Sep 23;20(9):e1012547. doi: 10.1371/journal.ppat.1012547 (PMC11449303; doi:10.1371/journal.ppat.1012547)
Supplement: S3 Table — (DOCX) [file ppat.1012547.s003.docx]

**S3 Table. Comparison of circulating biomarkers among Not Suppressed (NS)-, Late Suppressed (LS)- and Early Suppressed (ES)-PHIVAYA**

| **Parameters**  **Median [IQR]** | **(A)**  **NS-PHIVAYA**  **(N=14)** | **(B)**  **LS-PHIVAYA**  **(N=35)** | **(C)**  **ES-PHIVAYA**  **(N=6)** | **p-value***  **A *vs* B** | **p-value***  **A *vs* C** | **p-value***  **B *vs* C** |
| --- | --- | --- | --- | --- | --- | --- |
| 16S rDNA copies/µl | 105 [60-174] | 31 [11-91] | 15 [12-17] | **0.001** | **0.000** | 0.077 |
| mtDNA copies/µl | 467 [305-3122] | 148 [64.5-389] | 272 [249-343] | **0.000** | **0.000** | **0.005** |
| IL-6 pg/ml | 2.4 [1.7-3.8] | 1.2 [0.7-1.5] | 1.0 [0.8-1.3] | **0.000** | **0.000** | 0.186 |
| IL-8 pg/ml | 2.8 [2.3-7.6] | 3.1 [1.4-4.2] | 1.3 [1.1-2.0] | **0.004** | 0.087 | 0.120 |
| TNF-α pg/ml | 3.5 [3.2-5.7] | 3.4 [2.3-3.7] | 2.9 [2.5-3.0] | 0.629 | **0.025** | 0.524 |
| NCAM1 ng/mL | 684 [585-890] | 369 [287-601] | 339 [273-511] | **0.006** | **0.000** | 0.535 |
| CAF pg/mL | 2437 [1806-3133] | 2399 [1977-2990] | 2129 [2113-2165] | 0.748 | **0.039** | **0.001** |

**Adjusted by age, time on ART and time of ART initiation.*
