# Supplementary material for: HIV reservoir and premature aging: risk factors for aging-associated illnesses in adolescents and young adults with perinatally acquired HIV
Source: PLoS Pathog. 2024 Sep 23;20(9):e1012547. doi: 10.1371/journal.ppat.1012547 (PMC11449303; doi:10.1371/journal.ppat.1012547)
Supplement: S4 Table — (DOCX) [file ppat.1012547.s004.docx]

**S4 Table. Comparison of multifaceted aging biomarkers between PHIVAYA with comorbidities compared to PHIVAYA without comorbidities**

| **Parameters**  **median [IQR]** | **with comorbidities**  **(N=22)** | **without comorbidities (N=33)** | **p-value*** |
| --- | --- | --- | --- |
| %CD4+ activation | 1.8 [1.1-3.5] | 1.3 [1.0-1.7] | 0.327 |
| %CD8+ activation | 1.6 [1.0-2.7] | 1.0 [0.5-1.9] | 0.081 |
| %B activation | 8.3 [6.7-10.6] | 7.7 [5.6-11.3] | 0.905 |
| % CD4+ senescence | 10.0 [5.9-27.0] | 10.6 [5.6-19.1] | 0.553 |
| % CD8+ senescence | 6.5 [3.8-11.1] | 4.2 [2.9-12.5] | 0.194 |
| % B senescence | 9.6 [7.0-14.8] | 10.9 [9.1-14.6] | 0.640 |
| %CD4+ TIGIT+ | 3.7 [3.0-8.1] | 3.9 [2.0-5.5] | 0.443 |
| % CD8+ TIGIT+ | 17.0 [13.9-31.4] | 17.5 [12.3-26.8] | 0.707 |
| % T-regs | 13.6 [9.6-19.8] | 11.5 [10.0-18.4] | 0.968 |
| % B-regs | 3.6 [2.9-5.1] | 3.9 [2.6-4.8] | 0.551 |
| TREC copies10^6^/cells | 283 [119-390] | 435 [249-723] | 0.181 |
| RTL | 1.2 [1.1-1.3] | 1.2 [1.1-1.2] | 0.534 |
| 16S rDNA copies/µl | 92 [32-106] | 11 [9-17] | **0.000** |
| mtDNA copies/µl | 228 [96-498] | 249 [80-510] | 0.779 |
| IL-6 pg/ml | 1.4 [0.8-2.2] | 1.1 [0.7-1.3] | 0.190 |
| IL-8 pg/ml | 3.0 [2.4-4.3] | 2.0 [1.3-3.8] | 0.430 |
| TNF-α pg/ml | 3.4 [2.6-3.7] | 2.4 [2.2-3.4] | **0.011** |
| NCAM1 ng/ml | 466 [272-586] | 550 [329-729] | 0.762 |
| CAF pg/ml | 2665 [2155-3232] | 2165 [1746-2399] | **0.000** |

** Adjusted by age, HIV-DNA, time on ART and time of ART initiation*.
