# Supplementary material for: HIV reservoir and premature aging: risk factors for aging-associated illnesses in adolescents and young adults with perinatally acquired HIV
Source: PLoS Pathog. 2024 Sep 23;20(9):e1012547. doi: 10.1371/journal.ppat.1012547 (PMC11449303; doi:10.1371/journal.ppat.1012547)
Supplement: S5 Table — (DOCX) [file ppat.1012547.s005.docx]

**S5 Table. Comparison of multifaceted aging biomarkers between all PHIVAYA and each PHIVAYA subgroups with healthy controls**

| **Parameters**  **Median [IQR]** | **PHIVAYA**  **(N=55)** | **NS-PHIVAYA**  **(N=14)*** | **LS-PHIVAYA**  **(N=35)*** | **ES-PHIVAYA**  **(N=6)*** | **HEALTHY**  **CONTROLS**  **(N=23)** |
| --- | --- | --- | --- | --- | --- |
| %CD4 | 35.6 [28.2-38.9] | **30.9 [24.4-135.5]** | 36.9 [28.6-39.9] | 37.9 [36.4-38.7] | 36.6 [34.7-40.2] |
|  | 0.088 | **0.001** | 0.421 | 0.978 |  |
| %CD8 | **31.7 [25.3-40.2]** | **36.8 [31.2-42.2]** | **33.0 [24.8-40.2]** | 26.9 [20.2-30.0] | 21.1 [18.0-25.3] |
|  | **0.000** | **0.000** | **0.000** | 0.277 |  |
| CD4/CD8 | **1.0 [0.8-1.4]** | **0.8 [0.6-1.1]** | **1.0 [0.8-1.7]** | 1.4 [1.3-2.0] | 1.8 [1.4-2.2] |
|  | **0.000** | **0.000** | **0.000** | 0.272 |  |
| %CD4+ activation | **1.4 [1.0-2.5]** | **2.3 [1.3-3.1]** | **1.3 [1.0-2.2]** | **1.1 [1.0-1.4]** | 0.5 [0.4-0.6] |
|  | **0.000** | **0.000** | **0.000** | **0.000** |  |
| %CD8+ activation | **1.3 [0.7-2.1]** | **1.7 [0.7-2.5]** | **1.2 [0.8-1.8]** | 0.8 [0.3-1.4] | 0.4 [0.3-0.6] |
|  | **0.000** | **0.000** | **0.000** | 0.222 |  |
| %B activation | **8.0 [6.0-10.9]** | **9.7 [5.7-13.4]** | **8.3 [6.0-10.5]** | **6.9 [6.7-7.8]** | 3.4 [2.6-5.4] |
|  | **0.000** | **0.037** | **0.000** | **0.009** |  |
| % CD4+ senescence | **12.5 [5.9-22.2]** | **20.6 [16.9-27.9]** | **10.0 [5.3-21.4]** | 5.0 [3.4-7.5] | 3.7 [2.2-4.9] |
|  | **0.000** | **0.000** | **0.000** | 0.311 |  |
| % CD8+ senescence | **6.5 [3.4-12.8]** | **13.8 [7.5-17.4]** | **6.4 [3.1-12.3]** | 3.8 [3.3-5.2] | 3.4 [2.4-6.2] |
|  | **0.008** | **0.000** | **0.041** | 0.750 |  |
| % B senescence | 10.4 [7.2-14.6] | 9.1 [6.3-14.3] | 10.3 [8.3-14.6] | 12.0 [9.4-14.0] | 9.2 [4.8-11.8] |
|  | 0.087 | 0.544 | 0.071 | 0.129 |  |
| %CD4+ exhaustion | **3.9 [2.8-7.4]** | **5.3 [3.7-8.0]** | **3.8 [2.3-7.6]** | 2.8 [2.1-3.4] | 2.2 [1.0-3.9] |
|  | **0.001** | **0.000** | **0.001** | 0.333 |  |
| % CD8+ exhaustion | **17.8 [13.6-33.6]** | **41.6 [32.5-53.0]** | **16.0 [13.2-22.1]** | 12.0 [7.8-14.9] | 5.1 [3.9-7.6] |
|  | **0.000** | **0.000** | **0.000** | 0.102 |  |
| % T-regs | **11.8 [9.4-19.5]** | **11.6 [9.4-15.4]** | **12.2 [9.1-19.7]** | **15.1 [10.5-22.5]** | 4.2 [2.2-6.1] |
|  | **0.000** | **0.000** | **0.000** | **0.000** |  |
| % B-regs | **3.3 [2.4-4.4]** | **3.5 [2.5-4.1]** | **3.3 [2.4-4.2]** | **4.0 [3.2-4.9]** | 1.9 [0.9-2.9] |
|  | **0.001** | **0.033** | **0.005** | **0.001** |  |
| TREC copies/10^6^PBMC | **379 [213-692]** | **91 [38-251]** | 425 [280-884] | **505 [336-624]** | 750 [568-1506] |
|  | **0.000** | **0.000** | 0.068 | **0.005** |  |
| RTL | **1.2 [1.1-1.3]** | **1.1 [1.1-1.2]** | **1.2 [1.1-1.3]** | 1.3 [1.2-1.4] | 1.3 [1.2-1.4] |
|  | **0.011** | **0.000** | **0.044** | 0.942 |  |
| 16S rDNA copies/µl | **31 [15-105]** | **105 [60-174]** | **31 [11-91]** | 15 [12-17] | 6 [3-19] |
|  | **0.000** | **0.000** | **0.000** | 0.127 |  |
| mtDNA copies/µl | **258 [91-498]** | **467 [305-3122]** | **148 [65-389]** | **272 [249-343]** | 26 [6-91] |
|  | **0.000** | **0.000** | **0.000** | **0.000** |  |
| IL-6 pg/ml | **1.2 [0.7-1.7]** | **2.4 [1.7-3.8]** | **1.2 [0.7-1.5]** | **1.0 [0.8-1.3]** | 0.7 [0.5-1.0] |
|  | **0.000** | **0.000** | **0.008** | **0.047** |  |
| IL-8 pg/ml | **3.0 [1.4-4.1]** | **2.8 [2.3-7.6]** | **3.1 [1.4-4.2]** | 1.3 [1.1-2.0] | 2.1 [1.4-2.9] |
|  | **0.040** | **0.016** | **0.025** | 0.512 |  |
| TNFα pg/ml | 3.2 [2.3-3.6] | **3.5 [3.2-5.7]** | 3.4 [2.3-3.7] | 2.9 [2.5-3.0] | 2.6 [2.1-3.0] |
|  | 0.076 | **0.019** | 0.104 | 0.924 |  |
| NCAM1 ng/mL | **466 [287-694]** | **684 [585-890]** | **369 [287-601]** | 339 [273-511] | 283 [241-374] |
|  | **0.000** | **0.000** | **0.008** | 0.118 |  |
| CAF pg/mL | **2309 [1927-2907]** | 2437 [1806-3133] | **2399 [1977- 2990]** | 2129 [2113-2165] | 2120 [1663-2357] |
|  | **0.032** | 0.099 | **0.015** | 0.617 |  |

** p values were adjusted by age*
