# Supplementary figures and images for: HIV reservoir and premature aging: risk factors for aging-associated illnesses in adolescents and young adults with perinatally acquired HIV
Source: PLoS Pathog. 2024 Sep 23;20(9):e1012547. doi: 10.1371/journal.ppat.1012547 (PMC11449303; doi:10.1371/journal.ppat.1012547)

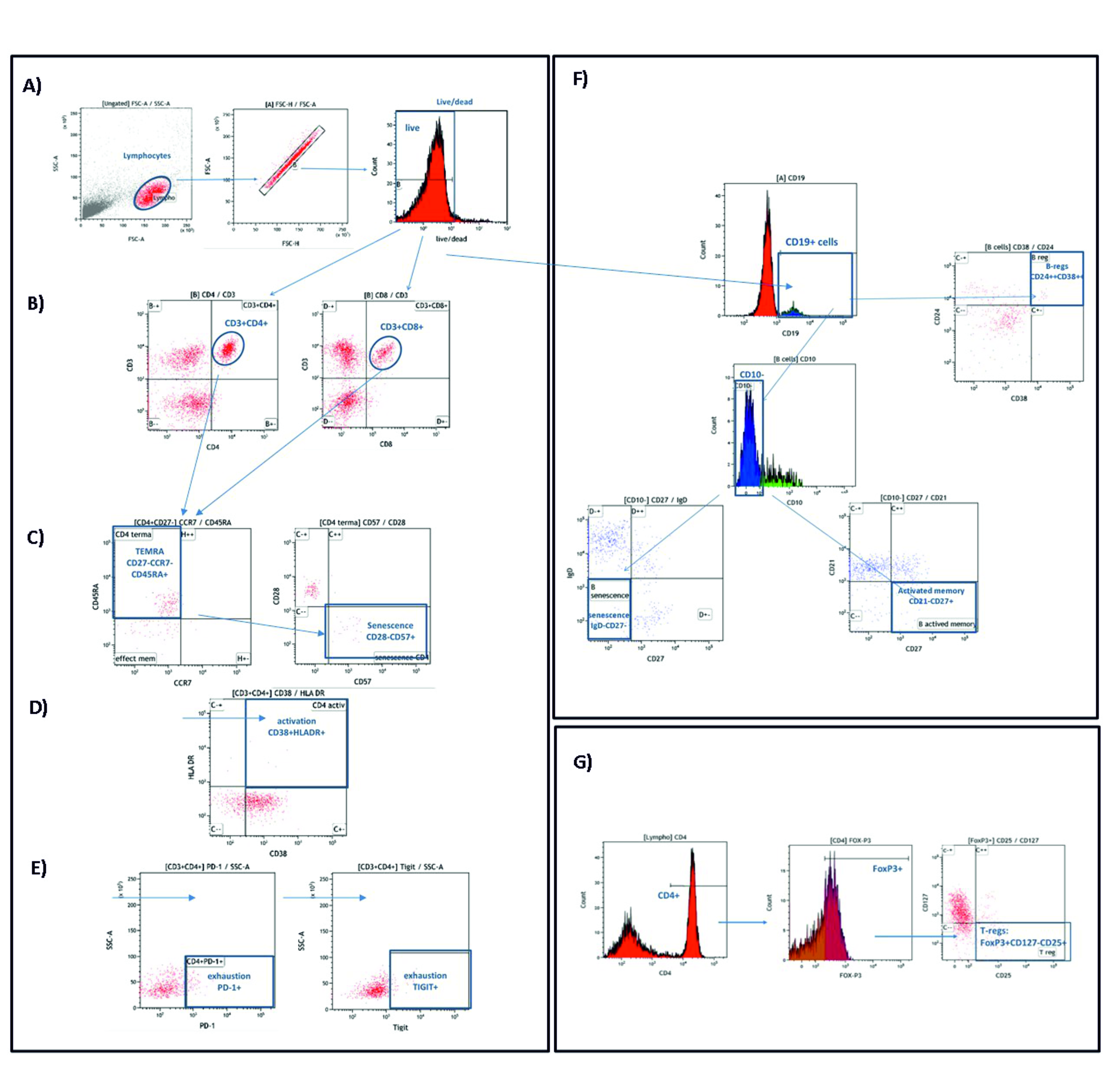

Supplement: S1 Fig — (TIF) [file ppat.1012547.s006.tif]
